# Impact of bioenergy feedstock carbon farming on sustainable aviation fuel viability in the United States

**DOI:** 10.1073/pnas.2312667120

**Published:** 2023-12-11

**Authors:** Sagar Gautam, Nawa Raj Baral, Umakant Mishra, Corinne D. Scown

**Affiliations:** ^a^Bioscience Division, Sandia National Laboratory, Livermore, CA 94550; ^b^Life-cycle, Economics, and Agronomy Division, Joint BioEnergy Institute, Lawrence Berkeley National Laboratory, Emeryville, CA 94608; ^c^Biological Systems and Engineering Division, Lawrence Berkeley National Laboratory, Berkeley, CA 94720; ^d^Energy Analysis and Environmental Impact Division, Lawrence Berkeley National Laboratory, Berkeley, CA 94720; ^e^Energy and Biosciences Institute, University of California, Berkeley, CA 94720

**Keywords:** aviation fuel, biomass, economic feasibility, SOC

## Abstract

Biomass-derived sustainable aviation fuel holds significant potential for decarbonizing the aviation sector. Its long-term viability depends on crop choice, longevity of soil organic carbon (SOC) sequestration, and the biomass-to-biojet fuel conversion efficiency. We explored the impact of fuel price and SOC value on viable biojet fuel production scale by integrating an agroecosystem model with a field-to-biojet fuel production process model for 1,4-dimethylcyclooctane (DMCO), a representative high-performance biojet fuel molecule, from Miscanthus, sorghum, and switchgrass. Assigning monetary value to SOC sequestration results in substantially different outcomes than an increased fuel selling price. If SOC accumulation is valued at $185/ton CO_2_, planting Miscanthus for conversion to DMCO would be economically cost-competitive across 66% of croplands across the continental United States (US) by 2050 if conventional jet fuel remains at $0.74/L (in 2020 US dollars). Cutting the SOC sequestration value in half reduces the viable area to 54% of cropland, and eliminating any payment for SOC shrinks the viable area to 16%. If future biojet fuel prices increase to $1.24/L-Jet A-equivalent, 48 to 58% of the total cultivated land in the United States could support a more diverse set of feedstocks including Miscanthus, sorghum, or switchgrass. Among these options, only 8–14% of the area would be suitable exclusively for Miscanthus cultivation. These findings highlight the intersection of natural solutions for carbon removal and the use of deep-rooted feedstocks for biofuels and biomanufacturing. The results underscore the need to establish clear and consistent values for SOC sequestration to enable the future bioeconomy.

## Significance

Cultivation of deep-rooted biomass crops can simultaneously achieve atmospheric carbon removal via SOC sequestration while providing much-needed energy-dense fuels to the aviation sector. These two outcomes may be incentivized differently. Farmers may be paid for SOC sequestration based on crop choice and farming practices, while biojet fuel can be sold at a premium on a volumetric basis without considering soil carbon impacts. However, price premiums for biojet fuels and payments for SOC sequestration will incentivize different activities, depending on the relative value. Combining agroecosystem and field-to-biofuel production process models reveals the likely outcomes of these two strategies and offers choices for what types of outcomes are preferable for the agriculture sector and the future sustainable aviation fuel industry.

Many countries are now prioritizing the establishment of commercial-scale biorefineries to reduce lifecycle greenhouse gas (GHG) emissions associated with transportation. In particular, the aviation sector is in need of renewable liquid fuels because air travel is difficult to decarbonize via other means such as battery-electric planes or fuel cells. The production and conversion of sustainable bioenergy crops into renewable fuels for aviation and other heavy-duty/marine applications offers promising opportunities for mitigating GHG emissions (1, 2). In the United States (US), the Department of Energy, Department of Transportation, and Department of Agriculture have committed to accelerating sustainable aviation fuel production, with a near-term target of 3 billion gallons per year by 2030 and up to 35 billion gallons per year by 2050 (3). Meeting these production goals necessitates parallel efforts to scale up low-input, sustainable biomass feedstock crops (4, 5). This strategy can yield both GHG mitigation in the aviation sector and near-term capture of carbon in the form of increased soil organic carbon (SOC) stocks.

In addition to meeting the need for renewable aviation fuels, large-scale cultivation of high-yielding biomass crops will also play a vital role in supporting the objectives of the “4 per 1000” initiative, launched at COP 21, which aims to increase the level of SOC in global cropland soils by an annual average of 0.4% (6). Selecting crops that both meet the needs of biorefineries and are tailored to maximize SOC sequestration for specific soil and site conditions will be essential. These crops should have the capacity to yield high biomass, sequester substantial amounts of atmospheric carbon, and thrive with minimal fertilizer and water inputs (7). The remaining question is the following: How should incentives be balanced between SOC sequestration and the production of low carbon-intensity biojet fuels? This study seeks to understand how placing different weight on SOC sequestration versus volume of biojet fuel production impacts the agricultural outcomes, both in terms of the available land and the diversity of crops that are economically viable to grow.

Future GHG emission scenarios that limit the mean global temperature to 1 °C or 2 °C above preindustrial era heavily rely on biomass energy with carbon capture and storage (8). However, studies rarely explore the interplay and tradeoffs between bioenergy and carbon removal at a country or global scale. There is a lack of national-scale research analyzing the performance of candidate bioenergy crops in different regions for biojet fuel production, despite the existence of numerous field-scale studies on bioenergy crops. Selecting the appropriate bioenergy crop, considering factors such as soil composition, weather patterns, topography, and existing infrastructure, is crucial to achieving an optimal feedstock supply with minimal emissions. Multiple studies have been conducted at the field scale to quantify biomass production and assess environmental impacts across various locations. Data gathered from these multilocation trials can be utilized to calibrate and benchmark agroecosystem models for different bioenergy crops. These models can then be employed to predict biomass productivity and assess environmental impacts at different spatial and temporal scales (9). By utilizing such models, we can gain insights into how SOC sequestration and biojet fuel incentives might shape the future biomass feedstock crop landscape in the United States.

There have been multiple studies conducted on individual bioenergy crops, investigating their SOC sequestration benefits and life cycle GHG emissions (10–14). A recent study by Uludere Aragon et al. (15) explored two perennial crops: switchgrass and Miscanthus. They focused primarily on marginal lands in the Eastern United States and used the ecosystem model Integrated Biosphere Simulator—agricultural version (Agro-IBIS) in combination with a mixed-integer optimization model to predict the scale of land conversion and biojet fuel production using a Fischer–Tropsch (FT) process across a range of GHG mitigation prices. In this study, we are specifically focused on understanding how two different levers (SOC sequestration value and biojet fuel selling price) impact the viability of biojet fuel production from sorghum, switchgrass, and Miscanthus on US agricultural lands. We accomplish this goal by integrating the simulation results from an agroecosystem model (DAYCENT) with an engineering model that includes supply chain costs and emissions as well as feedstock-specific conversion costs and emissions. Our representative biojet fuel molecule, 1,4-dimethylcyclooctane (DMCO), is a promising blendstock that can be produced from biomass through a hybrid biological-chemical process and provides a net heat of combustion up to 9.2% higher than Jet A (16). The study begins with the development of a spatially explicit database for biomass productivity and net emissions/sequestration for Miscanthus, sorghum, and switchgrass. We then incorporated fine-resolution crop yield and emissions/sequestration data into a technoeconomic analysis and life-cycle assessment framework to determine the minimum biojet fuel price (and carbon price) needed to justify bringing land on each grid cell into production as a biojet fuel feedstock.

## Results

### Miscanthus, Sorghum, and Switchgrass Biomass Productivity on Continental United States Croplands.

**Fig. 1.**
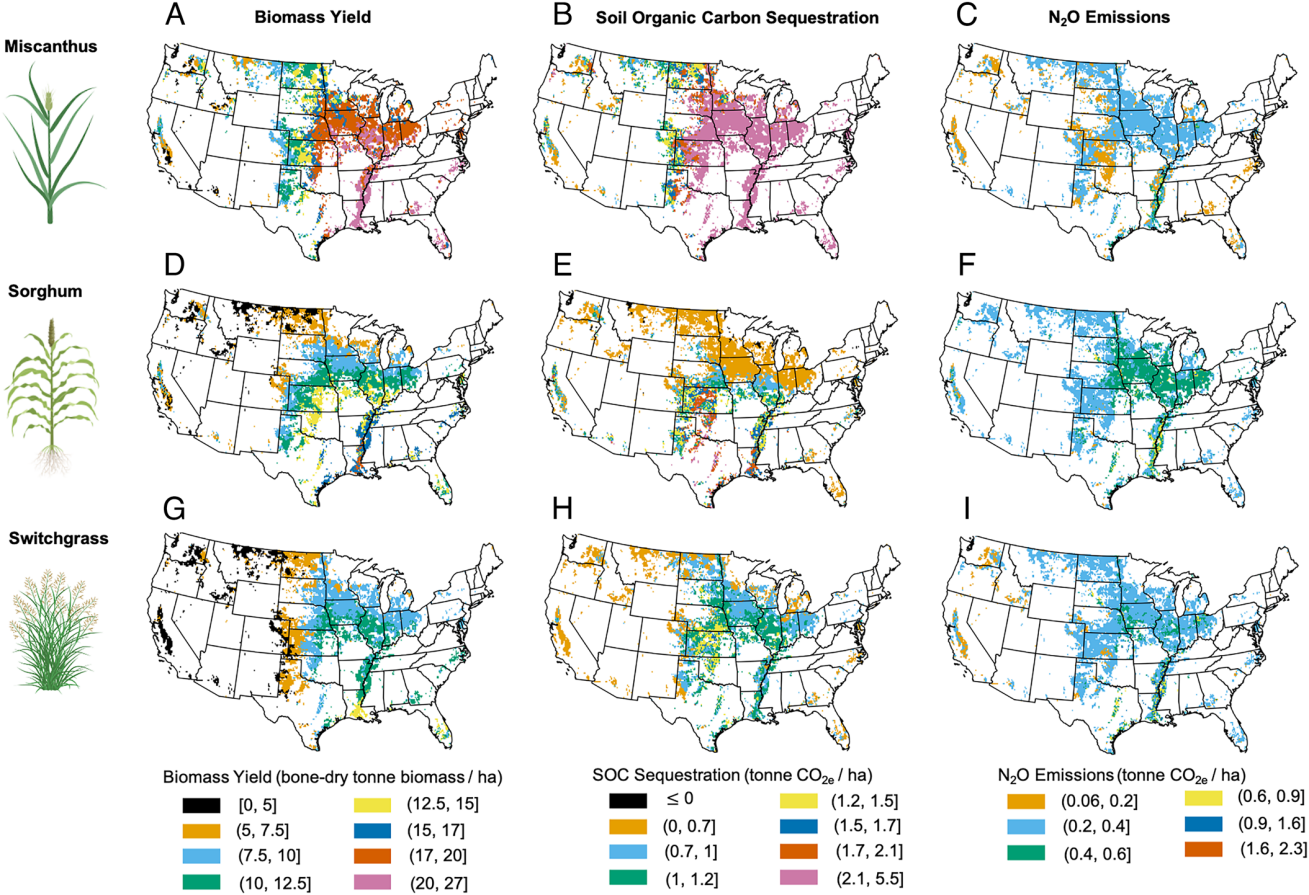
Biomass yield, soil organic carbon sequestration, and N_2_O emissions for continental United States croplands. Results for biomass Miscanthus (*A*–*C*), sorghum (*D*–*F*), and switchgrass (*G*–*I*) are presented in the *Top*, *Middle*, and *Bottom* panels, respectively.

An agroecosystem model (DAYCENT) was used to predict the biomass yield, SOC sequestration rate, and net GHG emission from continental United States cultivated lands under three different bioenergy crops (additional details on the modeling approach are provided in the *Materials and Methods*). Table 1 summarizes the average biomass yields. The simulated dry biomass yield for Miscanthus for continental United States ranged from 1.3 to 27 Mg ha^−1^ y^−1^, with spatiotemporal average of $14.6_{-4.2}^{+4.5}$   Mg ha^−1^ y^−1^ and a coefficient of variation of 37% (Fig. 1*A*). The range of simulated biomass yields including the first (Q1) and third (Q3) quartile based on decade long simulation result is presented in *SI Appendix*, Fig. S1. The sorghum results across continental United States shows dry biomass yield of 0.8 to 19.2 Mg ha^−1^ y^−1^, with spatiotemporal average of $9.7_{-2.4}^{+2.1}$   Mg ha^−1^ y^−1^ and a coefficient of variation of 35% (Fig. 1*D*). The details on modeling of sorghum can be found in Gautam et al. (10). The simulated dry biomass yield of switchgrass for continental United States ranged from 0.3 to 16 Mg ha^−1^ y^−1^, with a spatiotemporal average of $7.6_{-2.1}^{+2.3}$   Mg ha^−1^ y^−1^ and a coefficient of variation of 39% (Fig. 1*G*). The positive and negative values represent the interquartile range of prediction showing spatial and temporal variabilities in biomass yield. The range of simulated biomass yields including the first (Q1) and third (Q3) quartiles based on decade-long simulation results are presented in *SI Appendix*, Fig. S2. The comparison of multilocation and multiyear dry biomass yield observations with the model simulated results at observed sites resulted in root mean squared error (RMSE) of 3.1 Mg ha^−1^ y^−1^ and 5.8 Mg ha^−1^ y^−1^ for switchgrass and Miscanthus, respectively (*SI Appendix*, Fig. S3). The locations of experimental sites used for model verification are presented in *SI Appendix*, Fig. S4. The average observed and simulated biomass yields for switchgrass at five experimental sites were 7 Mg ha^−1^ y^−1^ and 9 Mg ha^−1^ y^−1^, respectively. The average observed and simulated biomass yields for Miscanthus at five experimental sites were 19 Mg ha^−1^ y^−1^ and 19.7 Mg ha^−1^ y^−1^, respectively. Results of the observed and simulated biomass yields across multiple years and locations for switchgrass and Miscanthus are presented in *SI Appendix*, Fig. S3. Across the continental United States, 17 million ha of cropland could produce Miscanthus biomass yields at or above 10 Mg ha^−1^ y^−1^, as compared to 6.2 million ha for switchgrass, and 10.2 million ha for biomass sorghum.

**Table 1.** Process modeling data inputs used to develop field-to-DMCO production model

Parameter	Unit		Miscanthus		Sorghum		Switchgrass
Lignocellulosic biomass feedstock							
Mean biomass yield^*^	bdt/ha		14.6		9.7		7.6
Mean SOC sequestration^*^	kg CO_2e_/ha		2.4		0.79		0.9
Mean N_2_O emission^*^	kg CO_2e_/ha		0.30		0.38		0.37
Biomass deconstruction (17, 18)							
Solid loading rate	wt%		30		30		30
Ionic liquid loading rate	g/g-biomass		0.125		0.125		0.125
Ionic liquid cost	$/kg		1		1		1
Enzyme loading rate	mg/g-glucan		10		10		10
Enzyme cost	$/kg-protein		4		4		4
Cellulose to glucose	wt%		95		95		95
Xylan to xylose	wt%		90		90		90
Bioconversion (19)							
Solid loading rate	wt%		25		25		25
Bioreactor power consumption	kW/m^3^		0.11		0.11		0.11
Bioconversion time	h		36		36		36
Glucose utilization	%		95		95		95
Xylose utilization	%		85		85		85
Recovery and separation (19)							
Recovery of isoprenol	%		98		98		98
Catalytic upgrading (19)							
Isoprenol to isoprene conversion rate	%		98		98		98
Dimerization catalyst loading rate	wt%		0.0013		0.0013		0.0013
Dimerization catalyst loading cost	$/kg		7.1		7.1		7.1
Isoprene to DMCOD isolated yield	%		98		98		98
Raney Ni catalyst loading	wt%		0.43		0.43		0.43
Raney Ni catalyst cost	$/kg		10.5		10.5		10.5
DMCOD to DMCO isolated yield	wt%		98		98		98

^*^Determined in this study. Detail results are documented in Fig. 1 and *SI Appendix*, Figs. S1– S6.

Maximum biological theoretical yield of isoprenol = 31.87 g/100 g of sugar, $1.5\;\text{Glucose}+2\;\text{Oxygen}=1\;\text{Isoprenol}+4\;{\text{CO}}_{2}+4\;H_{2}O$ , 1.8 Xylose+2 Oxygen= $1\;\text{Isoprenol}+4\;{\text{CO}}_{2}+4\;H_{2}O$ , SOC = Soil organic carbon; DMCOD = 1,6-dimethyl-1,5-cyclooctadiene; and DMCO = 1,4-Dimethylcyclooctane.

### Soil Carbon Changes and Nitrous Oxide Emissions.

The simulated baseline SOC stocks showed the RMSE of 1.9 kg m^−2^ and r^2^ of 0.43 based on comparison with the point observations of the rapid carbon assessment SOC database (10). Table 1 summarizes the average SOC changes and nitrous oxide emissions. The simulated SOC change under Miscanthus for the continental United States ranged from 0.15 to 5.5 Mg CO_2e_ ha^−1^ y^−1^ with a spatiotemporal mean of $2.4_{-0.7}^{+0.7}$   Mg CO_2e_ ha^−1^ y^−1^ (Fig. 1*B*). The simulated SOC change for sorghum for the continental United States ranged from −0.76 to 3.0 Mg CO_2e_ ha^−1^ y^−1^ with a spatiotemporal mean of $0.79_{-0.45}^{+0.38}$   Mg CO_2e_ ha^−1^ y^−1^ (Fig. 1*E*). The simulated SOC change under switchgrass for the continental United States ranged from 0.1 to 2.1 Mg CO_2_e ha^−1^ y^−1^ with a spatiotemporal mean of $0.9_{-0.16}^{+0.18}$   Mg CO_2e_ ha^−1^ y^−1^ (Fig. 1*H*). The average carbon sequestration for cropland areas with economic biomass yield in United States was 2.7, 1.2 and 1.1 Mg CO_2e_ ha^−1^ y^−1^, respectively for Miscanthus, sorghum and switchgrass bioenergy crops. The spatiotemporal mean of N_2_O emission for Miscanthus, sorghum, and switchgrass was $0.30_{-0.04}^{+0.03}$   , $0.38_{-0.06}^{+0.04}$   , and $0.37_{-0.07}^{+0.02}$   Mg CO_2e_ ha^−1^ y^−1^, respectively (Fig. 1 *C*, *F*, and *I*). The range of N_2_O emission predictions (Q1 and Q2) for Miscanthus and switchgrass are presented in *SI Appendix*, Figs. S5 and S6.

### Choice of Bioenergy Croplands Based on Biomass Yield and Soil Carbon Sequestration.

The ideal crop choice by location will depend, in part, on how net CO_2e_ sequestration is valued relative to biomass (and resulting biojet fuel) yield. The map in *SI Appendix*, Fig. S7 shows which crop achieves the highest biomass yield per ton of net CO_2e_ sequestration for each grid cell**.** Across eastern states, where rainfall is sufficient, Miscanthus out-performed switchgrass and sorghum. Sorghum was found to achieve the highest biomass yield per ton of net CO_2e_ sequestration in some of the lower western US states with lower mean annual precipitation and dry conditions, including croplands in Kansas and Texas. This is not surprising, given those regions already have higher cultivated acreage of sorghum in United States (20) (*SI Appendix*, Fig. S7). Switchgrass achieved the highest biomass yield per ton of net CO_2e_ sequestration in some locations in Kansas, Nebraska, and Montana (*SI Appendix*, Fig. S7).

### Choice of Bioenergy Croplands Based on Minimum Selling Price and Biofuel Carbon Footprint.

We developed separate techno-economic analysis (TEA) and life cycle assessment (LCA) models combining biomass production, supply, and the downstream biorefinery conversion processes for the three bioenergy crops: biomass Miscanthus, sorghum, and switchgrass. We considered DMCO as a representative renewable jet fuel molecule. For our study, we assumed each biorefinery sources feedstock from a circular feedstock collection area, where biomass feedstock is uniformly distributed around the biorefinery (*SI Appendix*, Fig. S8). The choice of the selected bioenergy crops in each 4-km square grid is guided by the impacts of their above-ground biomass yields, SOC sequestration/emissions, and N_2_O emissions on the production cost and carbon footprint of DMCO. For each grid cell, we modeled a biorefinery with uniform biomass feedstock yields, SOC sequestration, and N_2_O emissions within the feedstock collection area around the facility, aligning with the data from the individual grid cell. The resulting production costs and carbon footprints of DMCO for each bioenergy crop are presented in *SI Appendix*, Figs. S9–S11.

We assume a near-theoretical limit of the biomass-to-DMCO conversion rate, corresponding to a fully optimized process (Table 1). Without incorporating any value for GHG mitigation or SOC sequestration, we found that the production cost of DMCO ($/L-Jet A_eq_) in the continental United States falls in the range of 1.4 to 5.4 utilizing biomass sorghum, 1.2 to 3.2 utilizing Miscanthus, and 1.4 to 5.7 utilizing switchgrass feedstocks (*SI Appendix*, Figs. S9–S11). The variation within each biomass feedstock is due to the differences in its above-ground biomass yield across the United States (we do not attempt to predict variations in individual crop composition by location). The differences between the selected bioenergy crops are due to both the quality of biomass feedstock (determined by carbohydrates and lignin contents) and the above-ground biomass yields. While the carbohydrate content determines the amount of DMCO and the residual biomass, mainly lignin, alters the electricity generated onsite, the biomass yield is what dictates the biomass production and supply costs. For instance, the lower DMCO production cost with Miscanthus relative to biomass sorghum (*SI Appendix*, Figs. S9–S11) is not only due to its higher biomass yield but also 17% higher total carbohydrate content (Table 1).

The life-cycle GHG emission footprint of DMCO (g CO_2e_/MJ) in the continental United States falls in the range of 6.2 to 185.3 with biomass sorghum, 0.4 to 43.7 with Miscanthus, and −4.1 to 104.4 with switchgrass. In addition to the differences in the SOC sequestration and N_2_O emissions, the variation in carbon footprint across the selected bioenergy crops is impacted by differences in the biomass yield, total carbohydrate content, and lignin content. Higher carbohydrate content increases DMCO yields while high lignin content translates to more renewable fuel available for on-site electricity generation at biorefineries. For instance, switchgrass-derived DMCO achieves a lower net GHG footprint than Miscanthus in some locations because, despite its lower SOC sequestration potential, its 42% higher lignin content translates to a large carbon credit from the onsite electricity generation. In addition to lower GHG emissions credits generated from on-site electricity for biomass sorghum due to its lower lignin content, a higher GHG footprint for sorghum-derived DMCO relative to Miscanthus and switchgrass is driven by two other factors. First, we assume biomass sorghum is grown as an annual crop and requires fertilizer every year, while Miscanthus and switchgrass are perennial crops (with a lifetime of 20 y) that require a less average annual fertilizer. Second, biomass sorghum is hard to baling and drying down in the field relative to Miscanthus and switchgrass. This requires additional energy in the field for breaking hard stem of biomass sorghum, racking it in the field, and at least one time turning over the racked biomass.

The TEA results, prior to including GHG mitigation/sequestration values, highlight that biomass sorghum is most cost-effective as a feedstock in the southeastern United States, where the biorefinery could sell DMCO ($/L-Jet A_eq_) in the range of 1.05 to 1.58 (*SI Appendix*, Fig. S9). Miscanthus- and switchgrass-based biorefineries could sell DMCO at the same price range ($1.05 to 1.58 per L-Jet A_eq_) in the southeastern and midwestern regions of the United States. Notably, a Miscanthus-based biorefinery could sell DMCO at $1.05 to 1.58 per L-Jet A_eq_ in some parts of the northern and western United States (*SI Appendix*, Figs. S9–S11). The DMCO selling price could be more than $10/L-Jet A_eq_ in the northwestern United States with biomass sorghum, in the southern-California and south-western region of Arizona with Miscanthus, and in some parts of western United States with switchgrass.

Although the cost of DMCO production exceed that of conventional Jet A across all feedstocks, the selected bioenergy crops can contribute to substantial GHG emissions reductions (*SI Appendix*, Figs. S9–S11). We found that, in most parts of eastern and midwestern United States, biomass sorghum-based biorefineries could produce DMCO at a life-cycle GHG footprint 60 to 80% lower than Jet A. These emissions savings could increase by as much as 20% when DMCO is produced in the eastern and midwestern United States with either Miscanthus or switchgrass feedstocks. The GHG emissions reductions were diminished (to less than 60%) in the northern United States with biomass sorghum and in some parts of western United States with switchgrass. Miscanthus is the only feedstock in our study that could result in DMCO with >60% GHG footprint reduction relative to Jet A across the United States thus meeting the Renewable Fuel Standard’s threshold for cellulosic fuels.

**Fig. 2.**
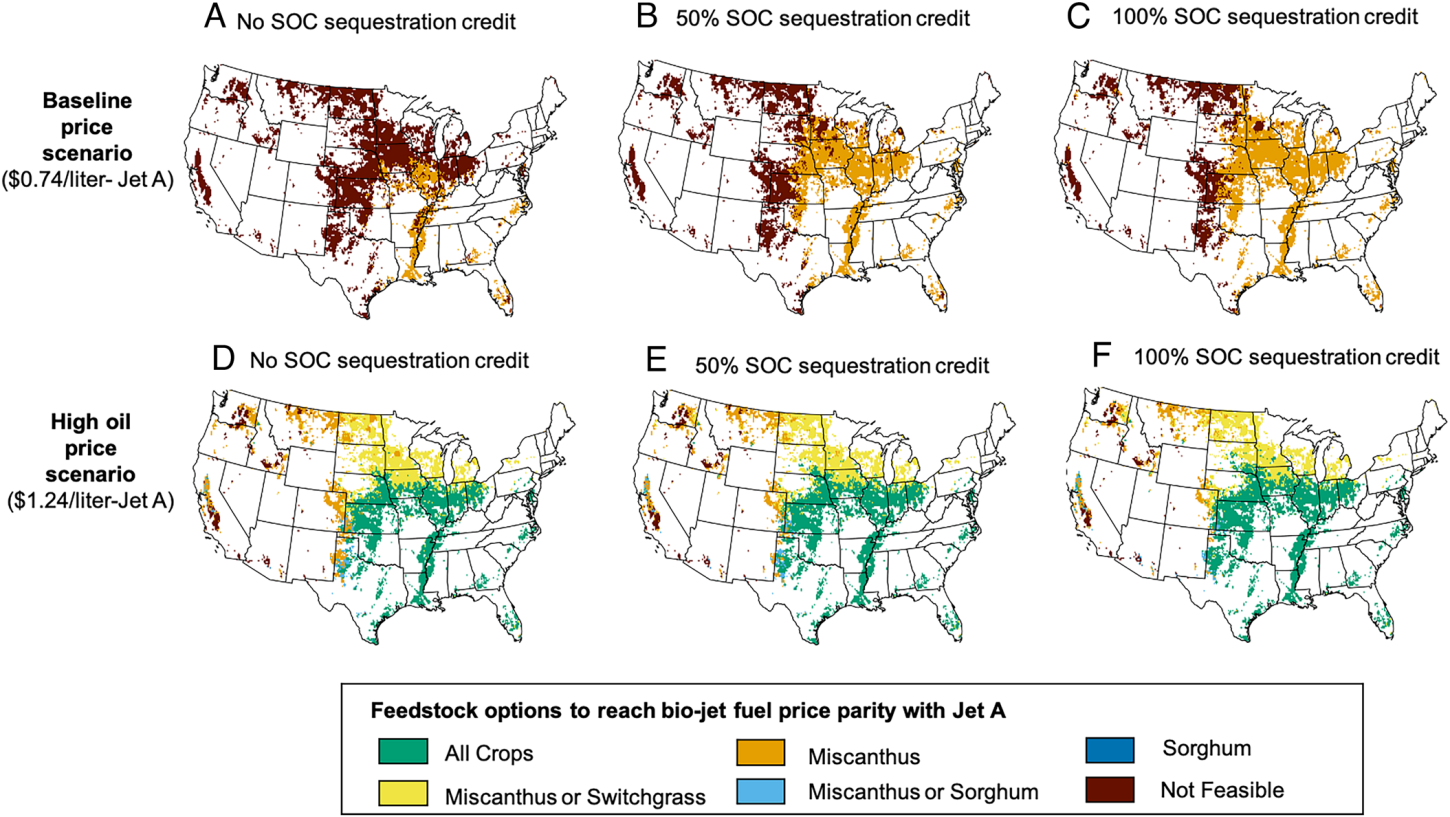
Bioenergy crops growing region across the US cultivated land for market-competitive bio-jet fuel production. Here, we considered different SOC sequestration credits (as a percentage of a social cost of $185/ton CO_2e_) and jet fuel selling prices. Left column (*A* and *D*): without any monetary credit for SOC sequestration; center column (*B* and *E*): 50% SOC sequestration valuation; and right column (*C* and *F*): 100% SOC sequestration valuation. Rows represent two different biojet fuel price scenarios.

To understand how different incentive structures might impact the viability of these three feedstocks, we combined the TEA, life-cycle GHG emissions assessment, and a social cost of GHG emissions reduction of $185/ton-CO_2e_ (21). The results offer insights into how biojet fuel selling prices and SOC sequestration credits impact the diversity of bioenergy crop options across the United States. We considered two different conventional jet fuel price scenarios: a baseline jet fuel price scenario ($0.74/L) and a high fuel price scenario ($1.24/L), respectively, corresponding to the conventional jet fuel prices in 2050 (2020$) with the reference case and high-oil-price scenario (22). Results highlight that only Miscanthus-based DMCO reaches cost-parity with the baseline conventional jet fuel price of $0.74/L, where Miscanthus can be economically grown on as much as 66.2% of the total United States cultivated land (Fig. 2 *A–C*). However, the value of carbon sequestration to soils has been questioned on the basis of its permanence (23). The overall DMCO GHG emissions footprint reduction due to the SOC sequestration could justifiably be assigned a discounted value relative to emissions avoidance or other more durable forms of carbon storage. Fig. 2 demonstrates the impacts of reducing the value of SOC sequestration. The economically feasible cultivated land for Miscanthus is reduced to 54.4% when the SOC credit is reduced by 50% and to 16.4% when no SOC credit is considered. These results indicate that even assigning 50% of the full $185/ton CO_2e_ to SOC sequestration (corresponding to $92.5/ton CO_2e_) is sufficient to ensure that Miscanthus as a biojet fuel feedstock will be a viable option for many US farmers. For the high-oil-price scenario, other selected bioenergy crops, including biomass sorghum and switchgrass, are also become feasible across the US cultivated land (Fig. 2 *A–C*). All the selected bioenergy crops are feasible on 58% of the US cultivated land when the SOC sequestration credit is fully included followed by 53.3% under 50% reduction carbon credit and 47.6% when the SOC credit is excluded (Fig. 2 *D–F*). For the high oil price scenario, as much as 97.2% of US cultivated land was found to be feasible for cultivation of bioenergy crops that result in DMCO selling price of <$1.24/L.

Regional electricity mixes do, and will continue to, impact the life-cycle GHG footprint of biojet fuel production across the US. DMCO is produced through a relatively electricity-intensive aerobic bioconversion process, but biorefineries also generate electricity by combusting lignin and other residual materials. Miscanthus’ higher lignin content means that Miscanthus-based DMCO production results in small net electricity exports, while sorghum and switchgrass-based facilities are net electricity consumers. The GHG footprint of the electricity grid in the United States has been declining due to rising share of renewable electricity such as solar and wind electricity and this reduces electricity offset credits for biorefineries that are net power exporters, while benefitting biorefineries that are net consumers. *SI Appendix*, Fig. S12 shows how grid decarbonization efforts alter the selection of bioenergy crops across US cultivated land. In all cases, the effects are small. For example, the fraction of viable cultivated land for Miscanthus at a minimum selling price at or below the baseline conventional jet fuel price in 2050 of $0.74/L (2020$) shrinks slightly from 64 to 62.7% when the grid is fully decarbonized (*SI Appendix*, Fig. S12).

## Discussion

We generated spatially explicit database of three bioenergy crops (Miscanthus, sorghum, and switchgrass) across cultivated lands in continental United states based on the productivity and environmental impacts. The productivity and GHG fluxes for these crops were modeled using an agroecosystem model. Additionally, we estimated the life cycle GHG emissions and cost of the biofuel production system by conducting life cycle and techno-economic analyses. Through the integration of agroecosystem modeling, TEA, and LCA, we identified viable locations for cultivating each of these bioenergy crops and quantified how varying weights on SOC sequestration might impact farmers’ crop choices.

Based on compiled data from 39 sites across the continental United States, switchgrass has been reported to frequently exhibit biomass yield ranging from 10 to 14 Mg ha^−1^ y^−1^. This range is influenced by factors such as cultivars, soil conditions, climate, and management practices (24). On the other hand, the rainfed productivity of Miscanthus has been reported to vary between 1 and 23 Mg h^−1^ y^−1^, with variability attributed to differences in rainfall, temperature, and solar radiation interception across various regions (13). The spatial distribution of biomass yield for both bioenergy crops is dependent on the local rainfall and temperature patterns. In the central and eastern US croplands with higher annual precipitation, both Miscanthus and switchgrass exhibit higher productivity. Conversely, in the western United States with drier conditions, productivity is lower. Previous studies have also noted a similar influence of precipitation on biomass yield for bioenergy sorghum (10, 13). Our results demonstrate a close relationship between temperature and the spatial distribution of biomass yield for bioenergy crops. This finding is consistent with earlier studies that reported a quadratic relationship between annual temperature and biomass yield across the continental United States (13, 24, 25).

The simulated SOC sequestration rates for Miscanthus, sorghum, and switchgrass in our study are consistent with experimental results observed in various regions of the United States (26–32). Current understanding of SOC dynamics suggests that cultivating perennial bioenergy crops, with increased below-ground carbon input and reduced soil disturbance, leads to higher soil carbon sequestration compared to annual crops (33). The lower loss of organic carbon from soil under perennial bioenergy crops can be attributed to the positive impact on soil fauna, which alters translocation and decomposition processes, thereby enhancing the carbon sequestration mechanism (34).

Our results show higher N_2_O emission from annual bioenergy crops, such as biomass sorghum, compared to the perennials such as Miscanthus and switchgrass. Among the selected perennial bioenergy crops, Miscanthus shows less N_2_O emission with a higher N-fertilizer application rate, indicating a higher nitrogen use efficiency when compared to switchgrass. Our results are consistent with experimental studies, which have also documented significantly higher nitrogen use efficiency of Miscanthus relative to switchgrass (35). An earlier study has also demonstrated that the large-scale conversion of annual crops to perennial bioenergy crops will significantly reduce GHG emissions and impacts the radiative forcing (36).

Miscanthus has been reported to have a higher water use efficiency per unit of biomass production compared to corn. It also has the ability to sequester SOC, resulting in a net carbon sink (37–39). On the other hand, sorghum is better suited for dryland regions when compared to other bioenergy crops due to its tolerance to drought and high temperatures. This tolerance is attributed to the accumulation of free proline in water-stressed sorghum leaves (40, 41). Ultimately, Miscanthus is advantaged relative to switchgrass and sorghum from an emissions standpoint in most regions due to its higher biomass yield potential and nitrogen use efficiency.

From a cost perspective, the choice of bioenergy crop is vitally important, as the biomass feedstock is responsible for about 44% of the DMCO production cost and more than half of the GHG footprint (before accounting for SOC sequestration). The SOC sequestration alone has the potential to reduce the GHG emissions footprint (all in g CO_2e_/MJ) of DMCO by 22.5 ± 14% for sorghum, 58.7 ± 10.8% for Miscanthus, and 54.5 ± 13.9% for switchgrass. Our results indicate a large variation in the SOC sequestration benefits across US croplands, meaning that careful selection followed by extensive measurement, reporting, and verification (MRV) will be crucial if SOC sequestration is more formally incorporated into GHG footprints for the purpose of incentivizing low-carbon biojet fuel production. This highlights the importance of a systems-level approach―integrating agroecosystem and field-to-biofuel production process models. Beyond selecting specific lands and commercial crops, there may be other climate- or soil-specific interventions that can boost SOC sequestration. For example, it would be beneficial to engineer bioenergy crops to increase biomass yield, carbon sequestration, and biomass quality. Utilizing smart/precision agricultural systems to maximize SOC sequestration and minimize N_2_O emissions can also be advantageous.

In terms of crop selection, our results indicate that Miscanthus shows promise as a bioenergy crop for economically viable sustainable aviation fuel production in the near term, particularly if a large value is placed on SOC sequestration but jet fuel prices remain lower. Other bioenergy crops such as biomass sorghum and switchgrass become more competitive when the price of crude oil increases. All three crops (Miscanthus, sorghum, and switchgrass) can produce cost-competitive biojet fuels on 58% of cultivated land in the United States in this high-oil-price scenario, while land suitable only for Miscanthus shrinks to just 8% of cultivated land. This suggests a possible need to balance a diverse and resilient agricultural system with the desire to maximize SOC sequestration that future biorefineries will have greater flexibility in choosing bioenergy crops.

The impact of SOC credits is highly nonlinear across the three cases we modeled. While offering a $92.5/ton CO_2e_ credit (50% of $185/ton CO_2e_) for SOC sequestration opens up a large portion of midwestern US lands for production, further increasing the credit from 50 to 100% has a comparatively small effect on the feasible land area. This is because SOC sequestration incentives have a limited impact in mitigating biomass feedstock costs, especially in regions with low biomass yields and for higher-input crops like annual biomass sorghum. Higher policy incentives will, of course, enhance the profitability of cultivating already feasible land. Our findings also highlight the fact that total biomass yield and on-field emissions/sequestration are not the only considerations. When considering the selection of feasible bioenergy crops for biofuel production, biomass yield, and biomass quality (as determined by carbohydrate and lignin contents) are important parameters.

Across all of our results, including biomass and conversion yields to DMCO, there are sources of uncertainty. Our predictions for biomass, SOC sequestration, and N_2_O emissions rely on field data in our calibrated DAYCENT model; this reduces some of the uncertainty in the delivered biomass cost and associated GHG emissions, although regional variations in some farming and transportation costs are not captured. Additionally, the DMCO conversion model is based on a fully optimized facility operating at commercial scale, extrapolating from smaller-scale experimental results. Our recent study (19) demonstrated a minimum selling price of DMCO at $9/L-Jet A_eq_, with 61.4 g CO_2e_/MJ of GHG emissions, considering biomass sorghum as the feedstock at the current state-of-the-art technology demonstrated at small scale. Once fully scaled and optimized, the cost and GHG footprint can reach $1.5/L-Jet A_eq_ and 18.3 g CO_2e_/MJ, respectively (Table 1). DMCO serves as a useful proxy in this study for a broader set of biojet fuels that can be produced through biological or hybrid biological-chemical means. While biorefineries should ideally be situated in close proximity to regional biomass resources, there is more flexibility in where liquid fuels can be transported. DMCO distribution can extend nationwide in the United States, with truck transportation costs estimated at $0.16 per metric ton per kilometer and associated GHG emissions at 0.008 g CO_2e_ per MJ per kilometer. These results emphasize the significance of regional DMCO production and distribution to prevent substantial additional costs and GHG emissions.

It is important to note that the bioenergy crops selected in this study have not been extensively cultivated across the continental United States, and therefore, the results are limited to validating models with small-scale experimental studies. Another limitation of this study is the constant phenotype representation, as there is a lack of spatial data to benchmark the model across different cultivars. The inclusion of commercial-scale agricultural management data, such as fertilizer application rates and biomass yield, could further improve the reliability, accuracy, and utility of our findings. Additionally, the adoption of genetically modified bioenergy crops could influence crop selection. There have been limited studies focused on modifying plant lignin in crops like Miscanthus (42), sorghum (43), and switchgrass (44), for example. However, it is possible that these plants could be modified in the future to reduce their recalcitrance during conversion to fuel and even increase their ability to sequester carbon in soils. As more field study data becomes available, such advances can be integrated into future studies. Note that while Miscanthus is native to eastern Asia, its unintentional spread is minimal due to its sterility as a hybrid (45). This sterility leads to the production of sterile seeds and requires rhizomes for propagation, which spread very slowly.

## Materials and Methods

We compared three bioenergy crops in this study. The Miscanthus and switchgrass simulations were conducted in this study. The sorghum simulation results were used from our previous study (11).

### DAYCENT Model.

We used the DAYCENT agroecosystem model (46) in this study. DAYCENT is a process-based model, which is the daily version of the monthly time step Century model (47). It is a one-dimensional model capable of simulating the fluxes of carbon and nitrogen and their cycling between different pools (atmosphere, soil, and vegetation) (48). The model includes submodels for plant productivity, decomposition of SOM and plant materials, greenhouse gases, and soil water and temperature dynamics. The input data required are weather (daily maximum/minimum temperature and precipitation as minimum requirement), site information (e.g., latitude), soil properties, crop types, and management. The major agricultural management practices and crop types can be represented in this model. The temperature, precipitation, and nutrient availability are the major environmental controls of biomass accumulation, SOM decomposition, and nitrogen fluxes. The model has been tested and applied in various agroecosystems and climate regions (49–51), including bioenergy systems (52–54).

### Study Area and Environmental Data.

The study area covered the croplands, pasture, and grasslands in the continental United States based on National Land Cover Database-2011 (55) hereafter cultivated lands. The weather data including daily minimum and maximum temperature and precipitation for continental United States was used from Global Historical Climatology Network (GHCN) datasets of National Centers for Environment Information datasets (56). Depth-wise soil properties datasets including the soil texture, hydraulic properties, bulk density, and pH were derived from the Soil Survey Geographic SSURGO datasets (57). For continental-scale assessment of the bioenergy crop, our model simulations were conducted in a 4-km grid; details about DAYCENT model setup can be found in Gautam et al. 2020.

### Model Setup and Identification of Bioenergy Combination.

The DAYCENT model was parameterized for each bioenergy crop based on the multilocation biomass productivity data for Miscanthus and switchgrass. Model parameter sensitivity was conducted using sensitivity package in R. The model was optimized to obtain the minimum root mean square error (RMSE) between observed and predicted biomass of the bioenergy corps. Baseline model for the existing cropping system and model for sorghum was used based on earlier DAYCENT model work on bioenergy crop by Gautam et al. (10). The details on the equilibrium and historic run for DAYCENT model can be found in Gautam et al. (10). The simulations for the two bioenergy crops of switchgrass and Miscanthus were conducted by simulations extended from the historical period of simulations. Each of the bioenergy crops was planted in 2008 to study the impact of decade-long cultivation of bioenergy crops. The assumptions for the large-scale cultivation of the bioenergy crops include a) rainfed conditions, b) biomass removal rate of 90%, and c) fertilizer application rate of 100 kg N ha for switchgrass and 120 kg N ha for Miscanthus. The rainfed assumption was used to find locations where bioenergy crops can be grown in water-limiting conditions (58). Although variability in crop yield is dependent on N fertilizer rate, earlier experimental studies suggested optimized yield of switchgrass with 100 kg N ha^−1^ (24, 59) and this fertilization rate is close to amount of nutrient removed (60). Similarly, for Miscanthus, the optimized yield was observed around 120 kg N ha^−1^ (14, 61).

Model parameterizations for each of the bioenergy crop were conducted by modifying the crop parameters in the DAYCENT model. The model parameter ranges were tested based on earlier DAYCENT model-based studies on switchgrass and Miscanthus; trial and error approach was used to match the observed range found in experimental sites to the model simulations (53, 62–64). The details on the model performance for the sorghum result used in this study can be found in Gautam et al. (10). For the model verification for each bioenergy crop, we used multiple bioenergy field trails study across continental United states for all three bioenergy crops. The model was calibrated using 7 y of data (2009 to 2015) of biomass yield from multiple field experiment for Miscanthus and switchgrass. Parameter included in model setup for Miscanthus and switchgrass including details about the selected parameter values are presented in *SI Appendix*, Table S1. The locations of the field trails used for the verification of the DAYCENT model are presented in *SI Appendix*, Fig. S1. The baseline model performance for SOC prediction was compared with observed RACA data for continental United states, detail of the model performance for SOC representation for baseline simulation can be found in Gautam et al. (10). The predicted maps of biomass yield, soil organic carbon change and nitrous oxide emission for each crop was compared pixelwise to identify the optimized locations. The bioenergy crop was assigned in each location based on the location with higher yield and net carbon sequestration.

### Technoeconomic Analysis and Lifecycle Assessment.

**Fig. 3.**
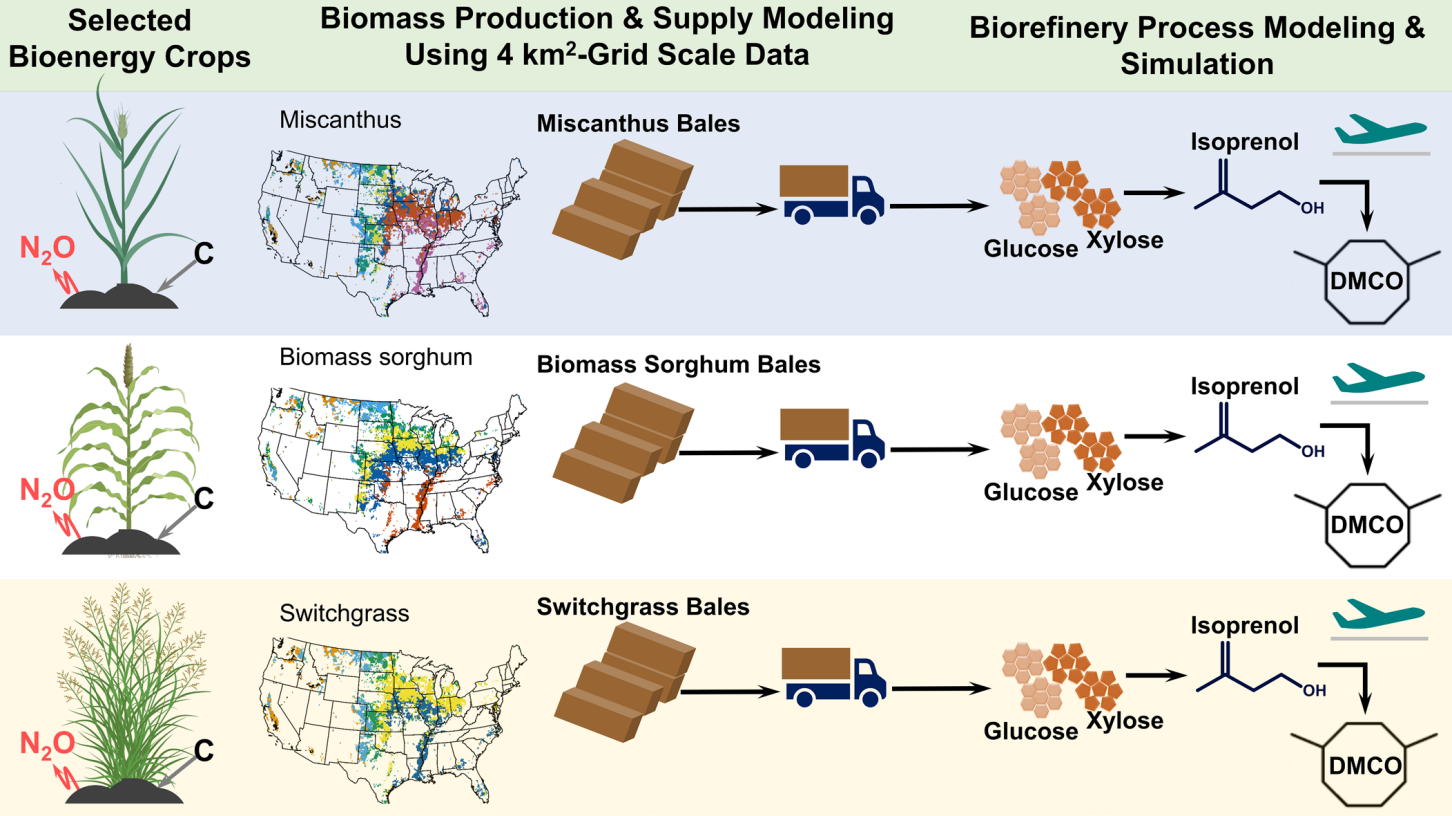
Overview of field-to-1,4-dimethylcyclooctane (DMCO) production system. DMCO is a cyclic alkane with a volumetric net heat of combustion up to 9.2% higher than Jet A (19).

The cost-effective and carbon-efficient bioenergy crop for a given location across the United States was determined considering the agroecosystem modeling results quantified in this study, including biomass yield, SOC sequestration, and N_2_O emissions, for the selected promising bioenergy crops, including biomass Miscanthus, sorghum, and switchgrass. The agroecosystem modeling results were incorporated in the field-to-DMCO―a high energy-density renewable jet fuel molecule with a volumetric net heat of combustion up to 9.2% higher than Jet A (19)―production model developed in this study (Fig. 3). For each bioenergy crop, the delivered biomass feedstock cost and associated lifecycle greenhouse gas (GHG) emissions at the biorefinery gate were determined considering the biomass yield determined at 4-km square-grid (Fig. 1 *A*, *D*, and *G*), where the biorefinery is assumed to be located at the center of the grid (*SI Appendix*, Fig. S8). The biorefinery sources the required amount of biomass feedstock to meet the scale of the biorefinery of 2,000 bone-dry metric ton (bdt)/day assuming that the biomass feedstock is uniformly distributed around the biorefinery. The SOC sequestration and N_2_O emission were further factored into the GHG emissions of the delivered biomass feedstock. The detailed mathematical expressions and data inputs used to determine biomass production and supply cost and associated GHG emissions are documented in prior studies (65–68).

Biomass is assumed to be delivered to the biorefinery in the form of bales (Fig. 3). The delivered biomass at the biorefinery goes through a series of subsequent conversion processes, including preprocessing (milling) to deconstruct biomass bales into 6.35 mm particles, ionic liquid-based biomass deconstruction to release simple sugars (primarily glucose and xylose), aerobic bioconversion to convert sugars into isoprenol, isoprenol recovery, and catalytic upgrading to convert isoprenol into DMCO. Full details of these conversion processes are documented in prior studies (19, 69). The biorefinery process model further includes wastewater treatment, onsite energy generation, and utility stages, which are consistent with prior studies (19, 70). Wastewater is treated with anaerobic and subsequent aerobic treatment processes. The onsite energy stage generates process steam and electricity utilizing the unutilized biomass (mainly lignin), the biogas generated from the anaerobic wastewater treatment, and the makeup natural gas. The makeup natural gas was added only if the onsite biogenic energy sources are not sufficient to meet the heat and electricity demands of the biorefinery. The utility stage provides process water, cooling water, and chilled water. Additionally, the utility stage includes the clean-in-place system, which provides on-demand cleaning solutions to the reactors and the distillation columns. Table 1 summarizes major data inputs used to develop the biorefinery process model. Additional biorefinery process data inputs are documented in *SI Appendix*, Table S2.

The rigorous field-to-biorefinery process simulation was carried out combining Macro-Enabled Microsoft Excel and process modeling software-SuperPro Designer. This simulation generates material and energy balances for each unit operation, which were used to determine its size, quantity, and purchase cost. The equipment purchase cost was used to determine installation and other direct and indirect costs. Consistent with previously published technoeconomic studies, we use the discounted cash flow rate of return (DCFROR) analysis to determine the minimum selling price of DMCO―the selling price that reduces the net present value to zero. Briefly, we assume an internal rate of return (IRR) after taxes of 10%, plant lifetime of 30 y, plant operating hours of 7,920 h (330 d/y and 24 h/d), and income tax of 21% (19, 70).

The life-cycle GHG footprint of DMCO was determined using a hybrid process-based/input–output-based life cycle inventory approach documented in previously published work (71). This LCA model takes in the material and energy balance results generated by SuperPro Designer and the Microsoft Excel–based biomass production and supply model. Then, the LCA model generates physical units-based input–output matrix, uses build-in matrix of the GHG impact vectors, and calculates the direct and indirect GHG emissions. The GHG impact vectors (*SI Appendix*, Table S3) were gathered from widely used LCA databases (72). The GHG emissions impact of electricity generated onsite and required for DMCO production processes was calculated by considering the carbon footprint of the subregional electricity mix as identified by USEPA (*SI Appendix*, Table S4 and Fig. S13). We accounted for excess electricity credits using the system expansion method, assuming that the excess electricity is exported to the subregional grid electricity mix. Detailed carbon footprints of US subregional electricity mixes are documented in *SI Appendix*, Table S4. We assumed the functional unit of 1 MJ (HHV) of DMCO. *SI Appendix*, Table S5 summarizes the mass density and the theoretically estimated higher heating values of DMCO. The carbon footprint reduction benefit was calculated considering carbon saving relative to conventional jet fuel and California’s low-carbon fuel standard credit of $193.2 per metric ton of CO_2e_-avoided (73).

## Supplementary Material

Appendix 01 (PDF)

## Data Availability

All study data are included in the article and/or *SI Appendix*.
