# Supplementary material for: Impact of bioenergy feedstock carbon farming on sustainable aviation fuel viability in the United States
Source: Proc Natl Acad Sci U S A. 2023 Dec 11;120(51):e2312667120. doi: 10.1073/pnas.2312667120 (PMC10742374; doi:10.1073/pnas.2312667120)
Supplement: Supplementary file 1 — Appendix 01 (PDF) [file pnas.2312667120.sapp.pdf]

Supporting Information

Impact of bioenergy feedstock carbon farming on sustainable aviation fuel viability in the United States

Sagar Gautam[a,b,1,*], Nawa Baral[b,c,1], Umakant Mishra[a,b], & Corinne D Scown[b,c,d,e]

[a] Bioscience Division, Sandia National Laboratory, Livermore, CA 94550, United States

[b] Joint BioEnergy Institute, Lawrence Berkeley National Laboratory, Emeryville, CA 94608, United States.

[c] Biological Systems and Engineering Division, Lawrence Berkeley National Laboratory, Berkeley, CA 94720, United States.

[d] Energy Analysis & Environmental Impact Division, Lawrence Berkeley National Laboratory, Berkeley, CA 94720, United States.

[e] Energy & Biosciences Institute, University of California, Berkeley, CA 94720, United States.

[1] These authors contributed equally.

Corresponding author: sgautam@sandia.gov

This file includes:
Supporting Figures S1 to S13
Tables S1 to S5
SI References

a

b

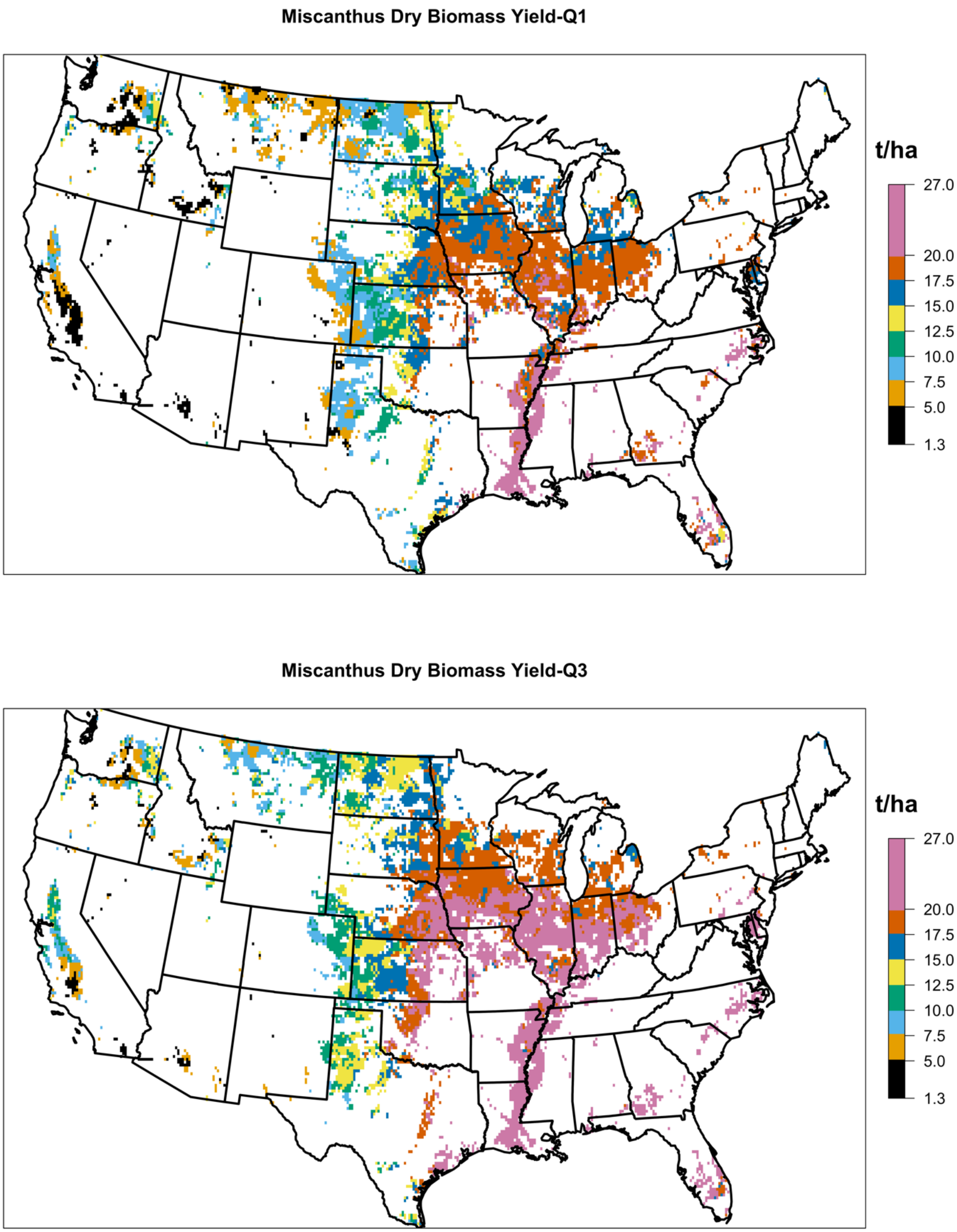

Fig. S1. The simulated rainfed biomass yield of miscanthus across continental United States using the Daily Century model a) first quartile (Q1) and b) Third quartile (Q3).

a

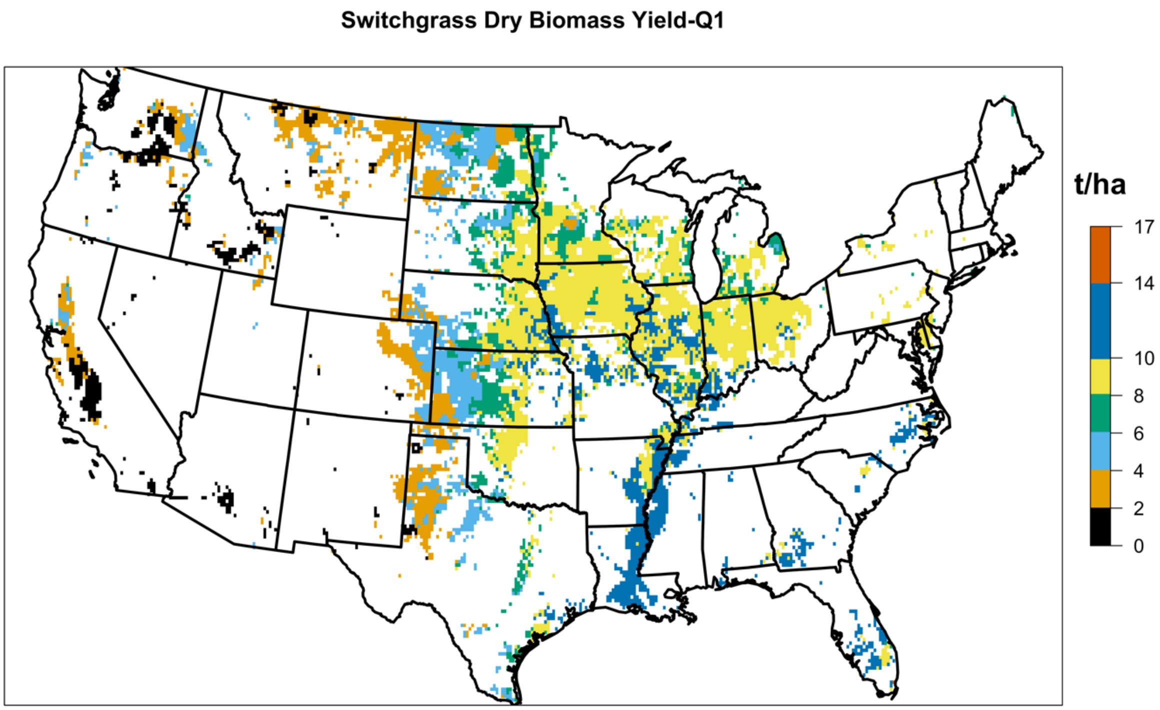

b

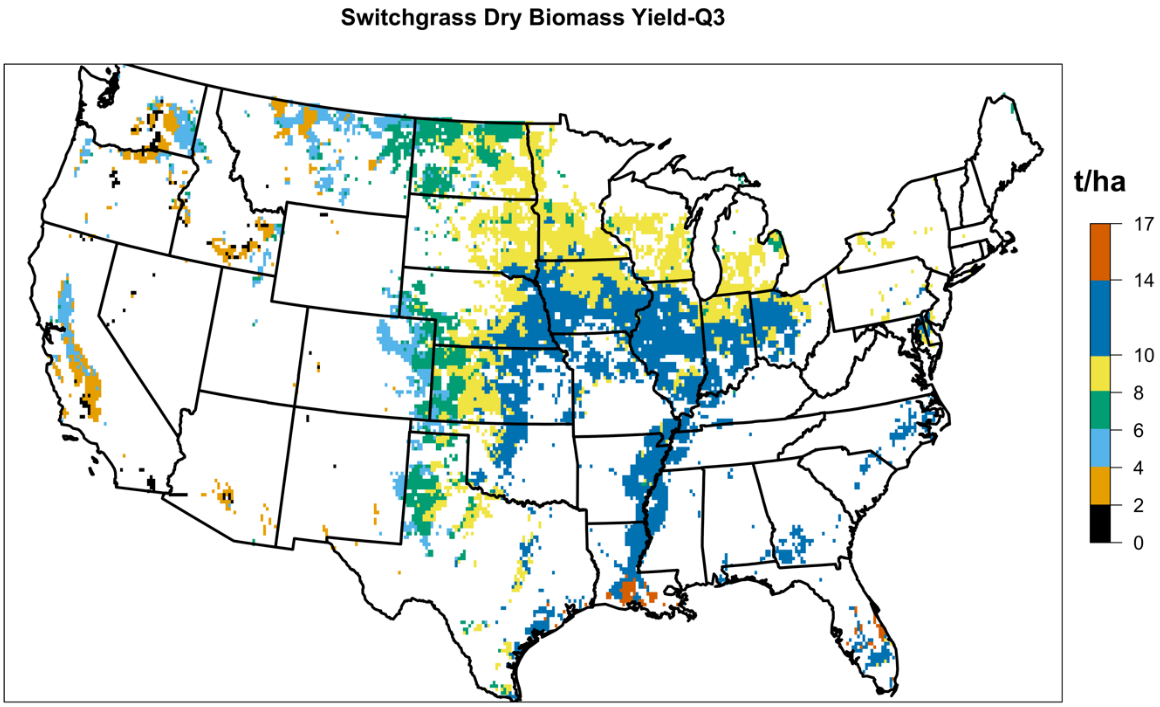

Fig. S2. The simulated rainfed biomass yield of switchgrass across continental United States using the Daily Century model a) first quartile (Q1) and b) Third quartile (Q3).

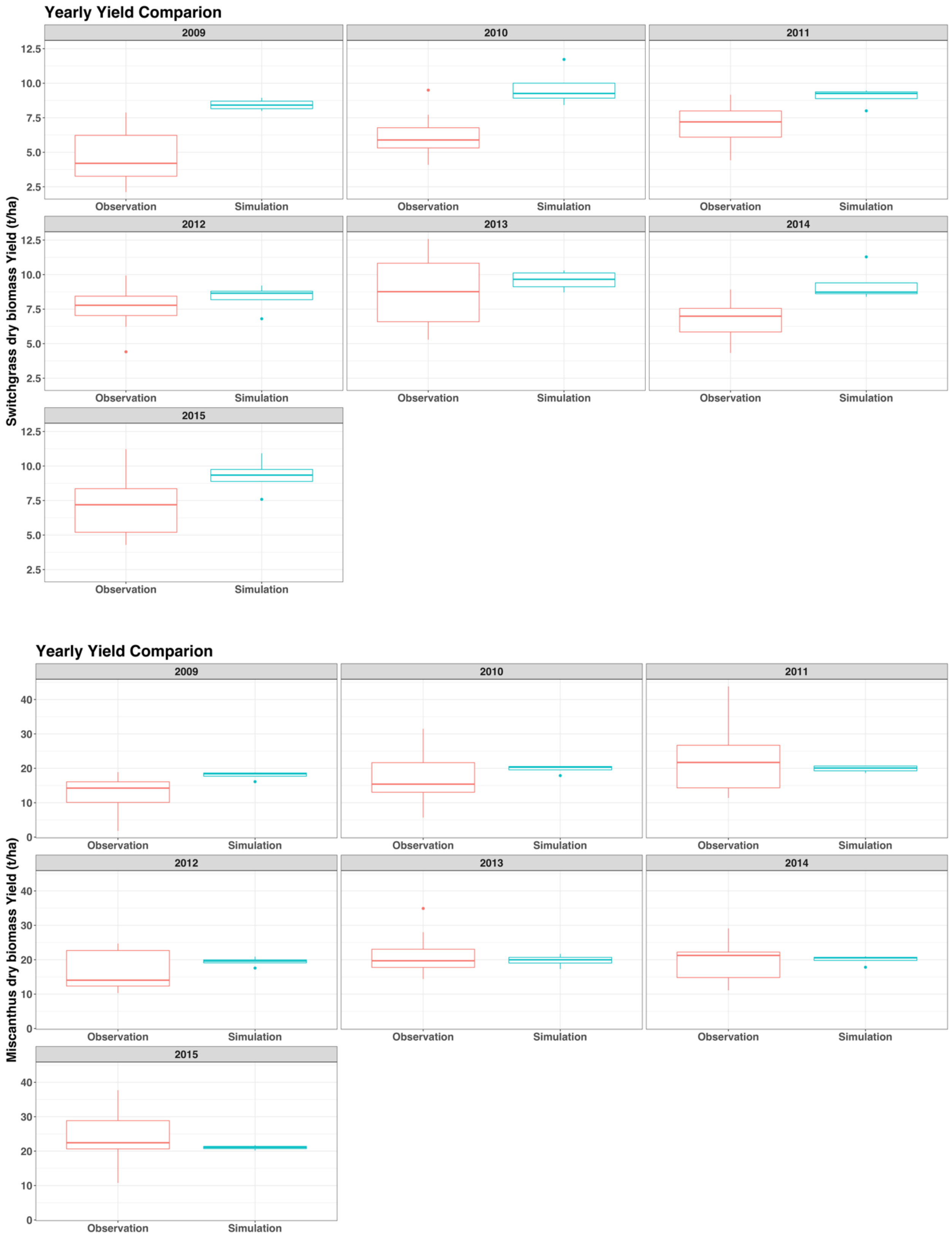

Fig. S3. The comparison of DAYCENT simulated and observed yield based on data collected from multiple location a) comparison for switchgrass and b) comparison for miscanthus.

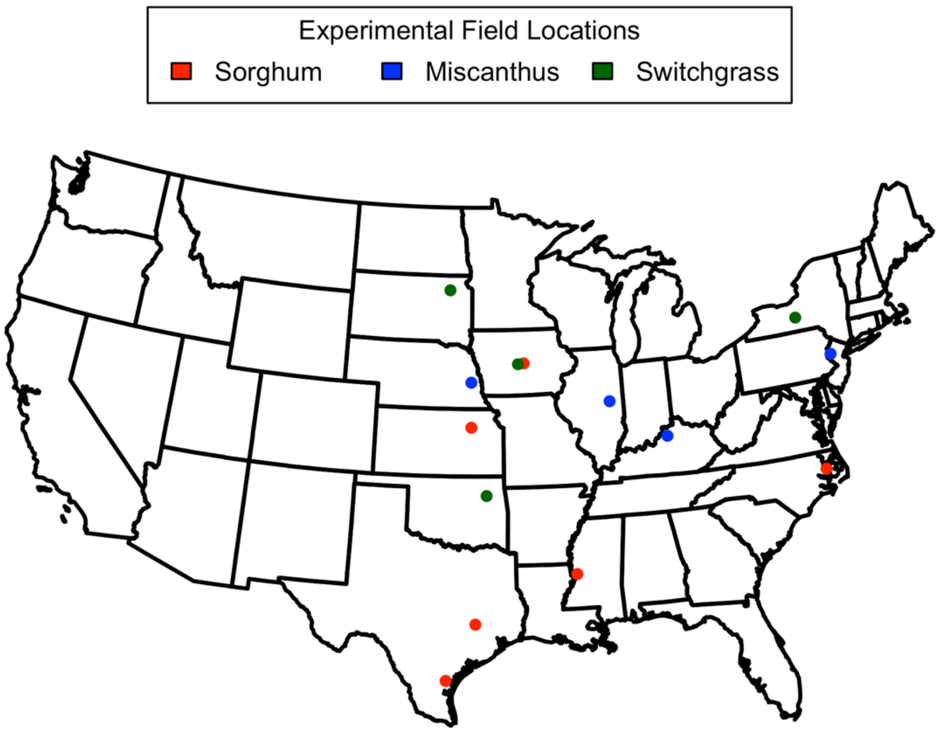

Fig. S4. Experimental field location different bioenergy crops used for model verification for sorghum, miscanthus and switchgrass

a

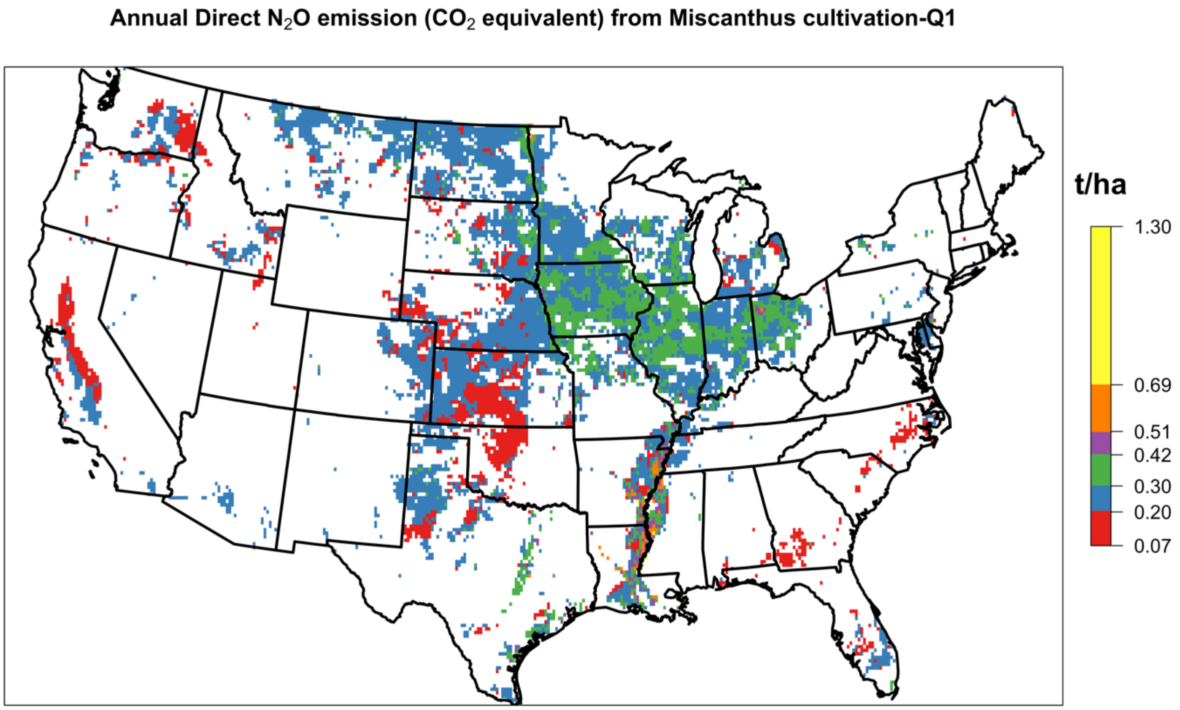

b

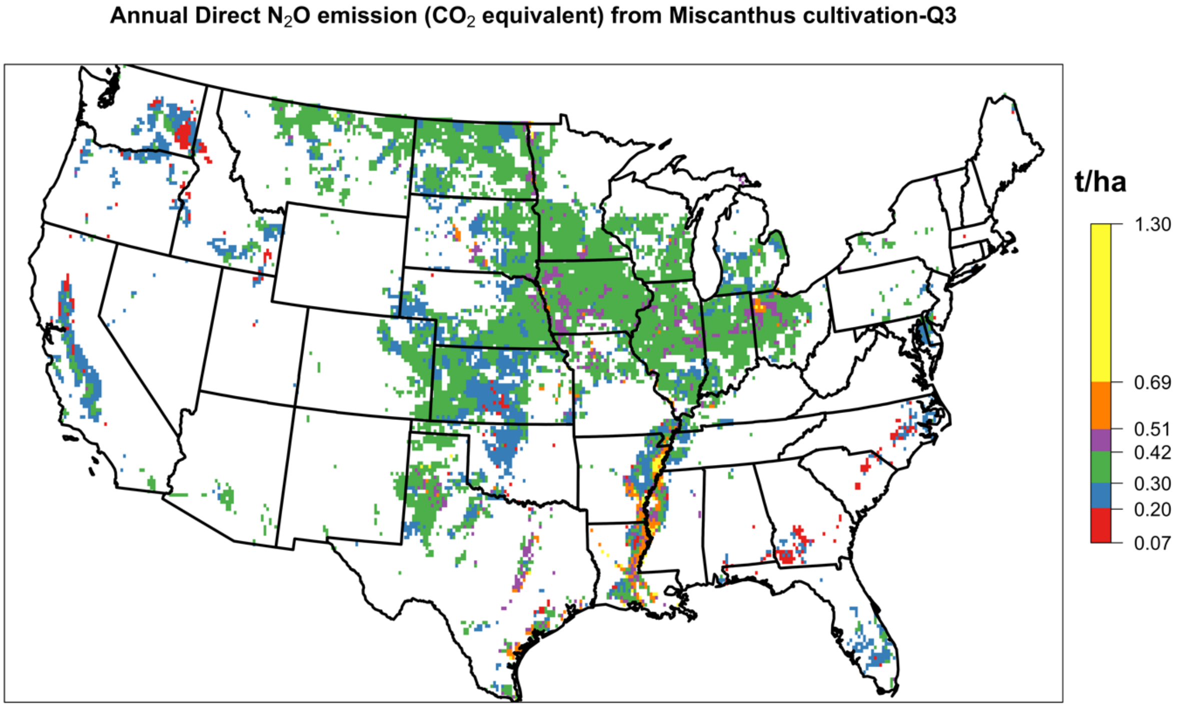

Fig S5. Simulated mean emission of direct nitrous oxide as carbon dioxide equivalent due to decade long cultivation of Miscanthus a) first quartile (Q1) and b) Third quartile (Q3).

a

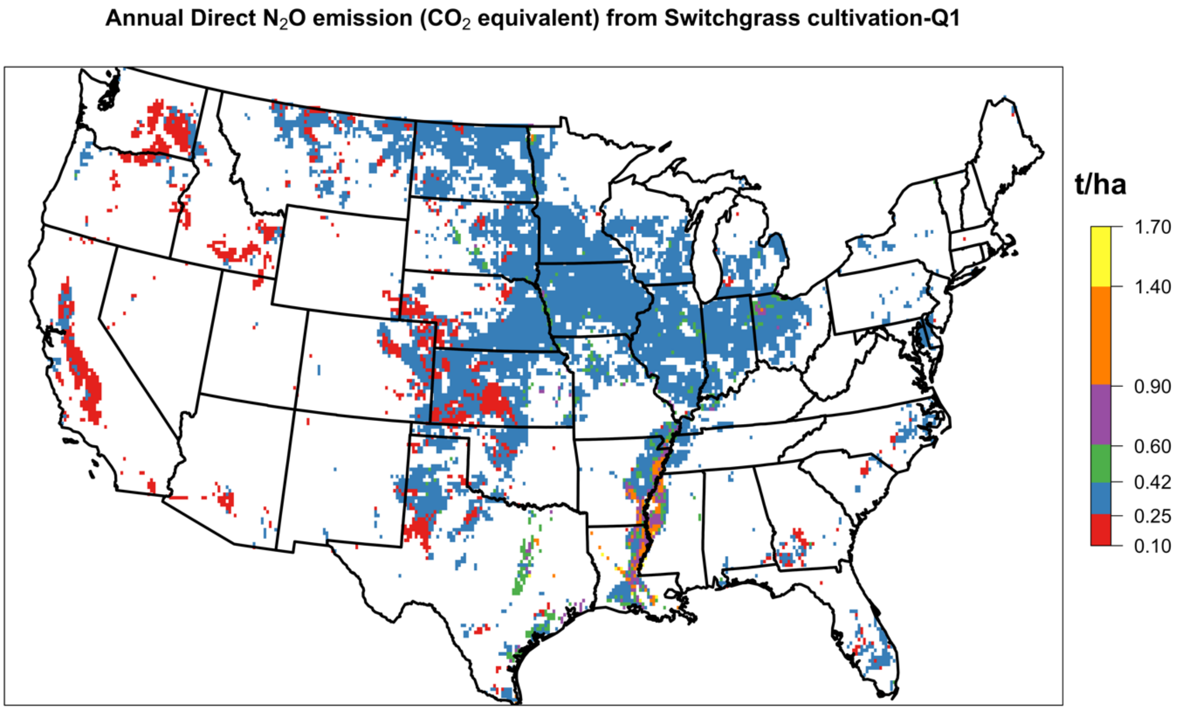

b

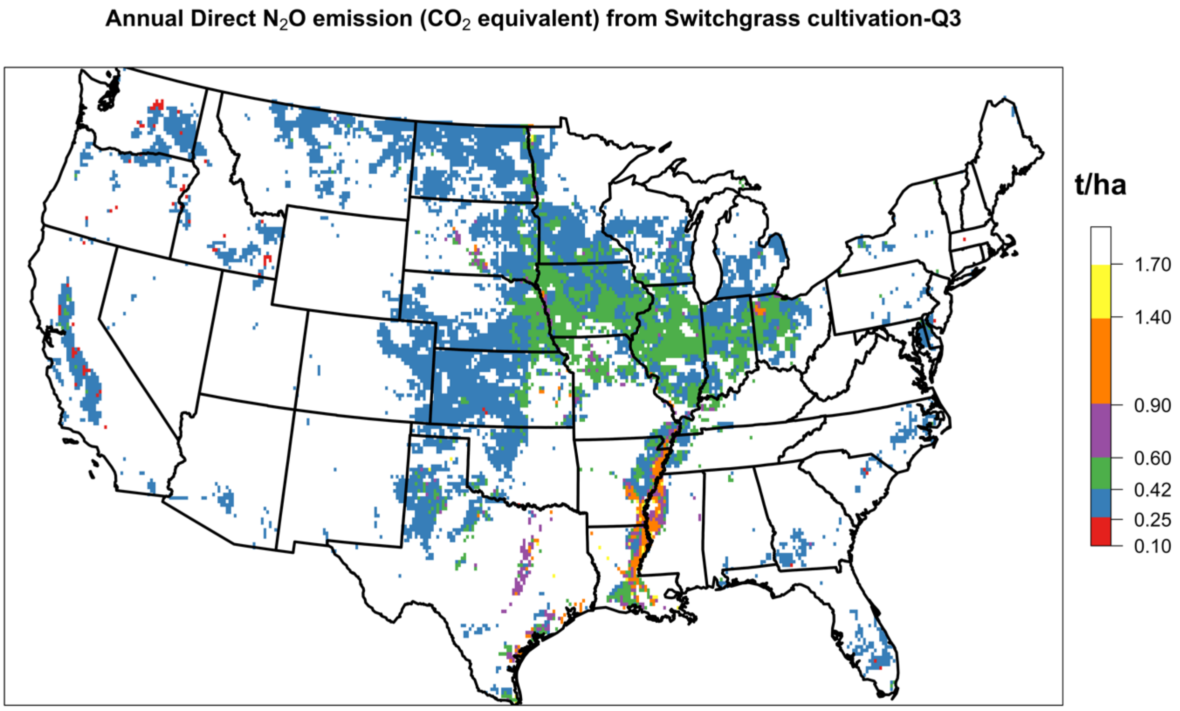

Fig S6. Simulated mean emission of direct nitrous oxide as carbon dioxide equivalent due to decade long cultivation of switchgrass a) first quartile (Q1) and b) Third quartile (Q3).

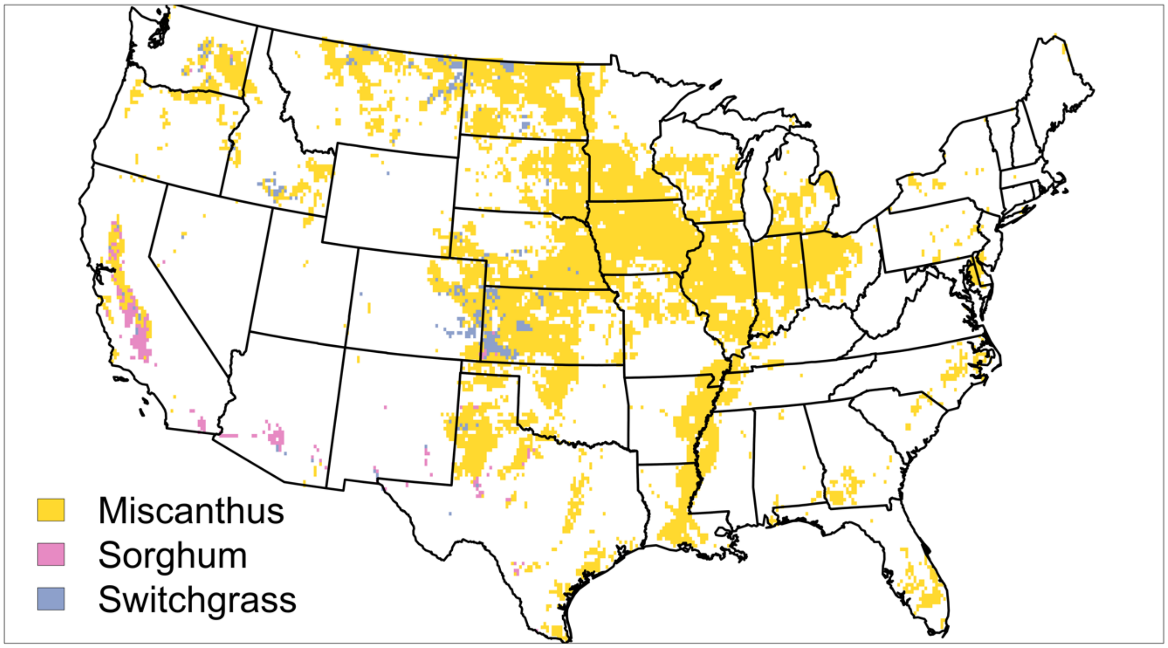

Fig S7. Cultivated lands of the continental United States that will produce maximum biomass yield per unit of net carbon sequestration (accounting for SOC sequestration and on-farm N_2O emissions); different color represents the favorable area found for the cultivation of each of three bioenergy crops compared in this study.

Biomass feedstock collection area and the location of a biorefinery

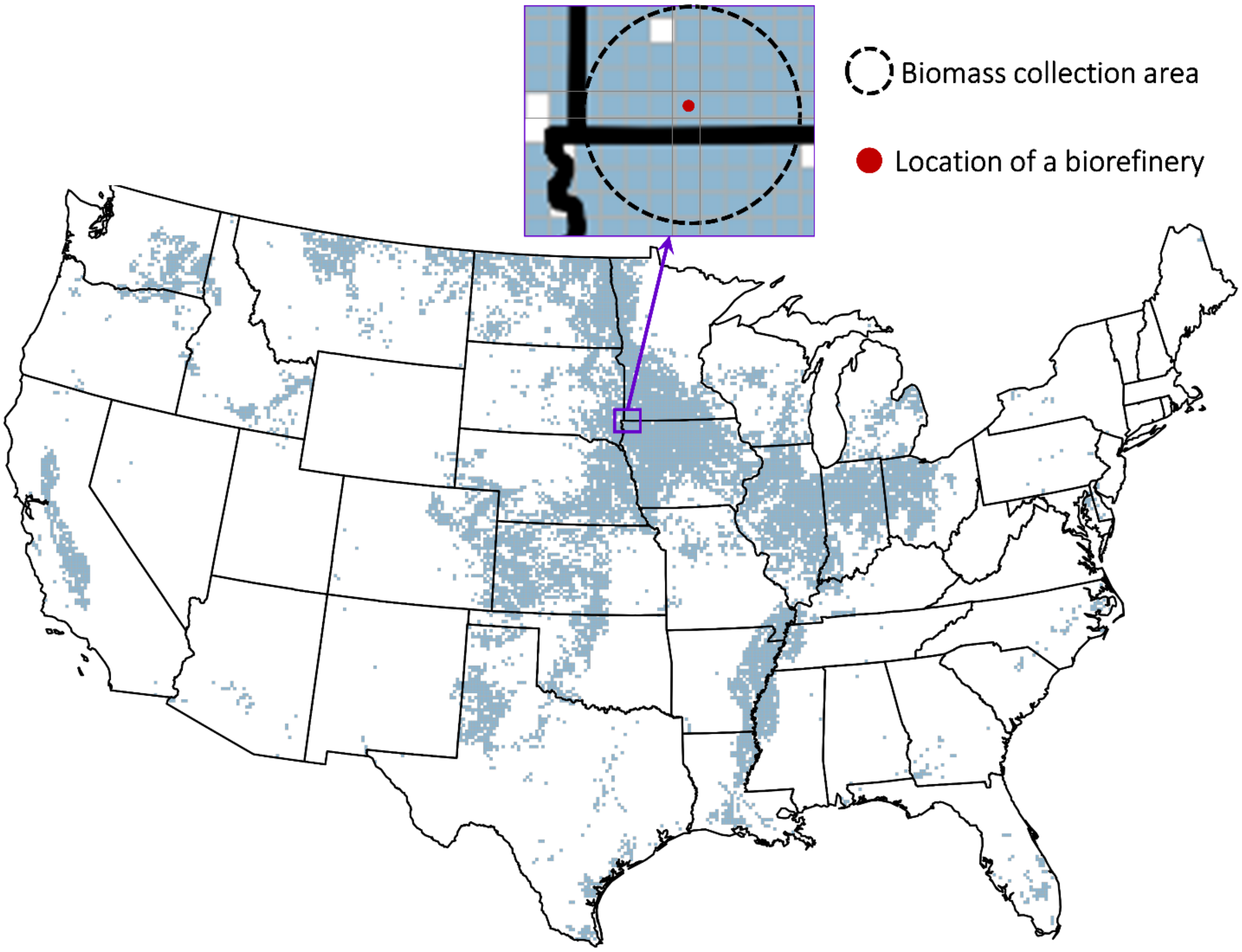

Fig. S8. An example of biorefinery location and the biomass feedstock collection area. The grid area shows potential bioenergy crops, including sorghum, miscanthus, and switchgrass, cultivation area.

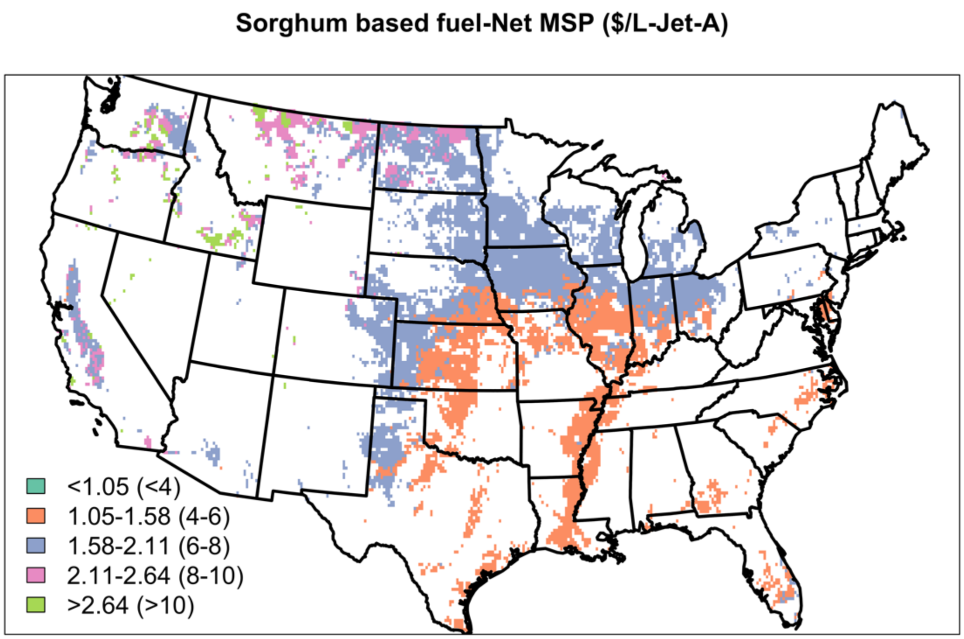

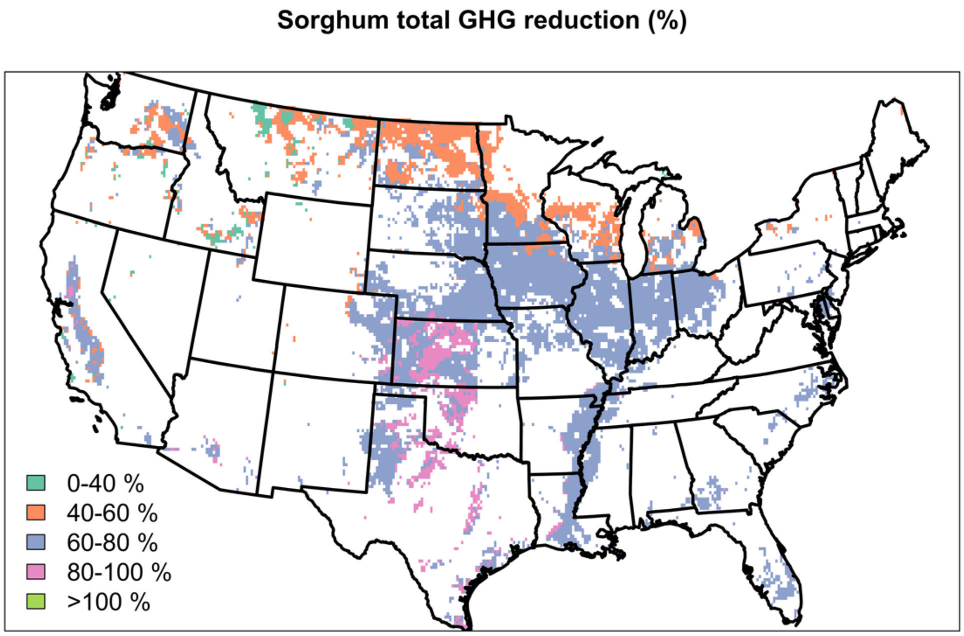

Fig. S9. Minimum selling price and carbon footprint of DMCO utilizing biomass sorghum. The percent reductions in the carbon footprint of DMCO were calculated relative to the carbon footprint of conventional Jet A (89 gCO_{2e}/MJ) (1).

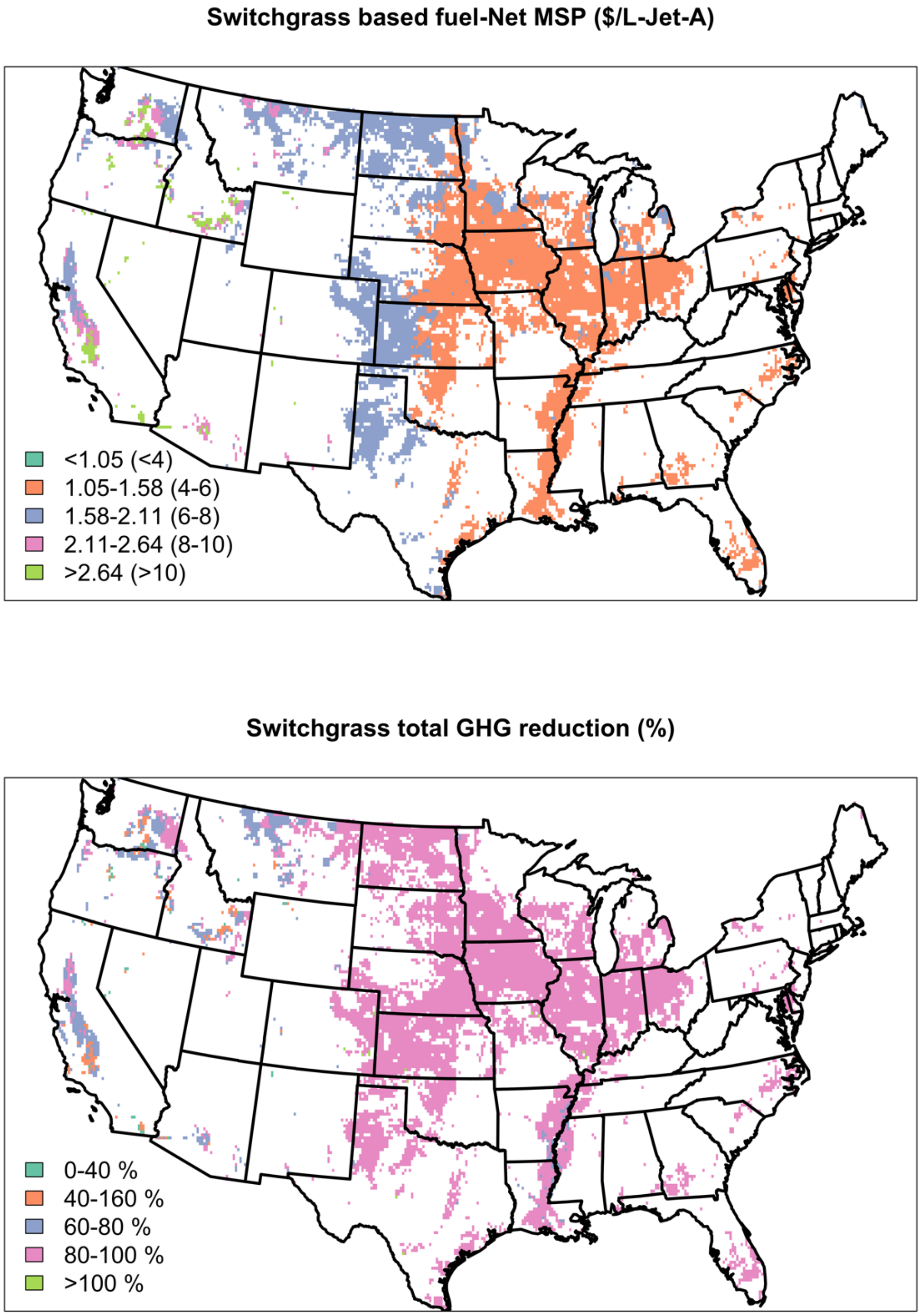

Fig. S10. Minimum selling price and carbon footprint of DMCO utilizing switchgrass. The percent reductions in the carbon footprint of DMCO were calculated relative to the carbon footprint of conventional jet fuel of 89 gCO_{2e}/MJ(1).

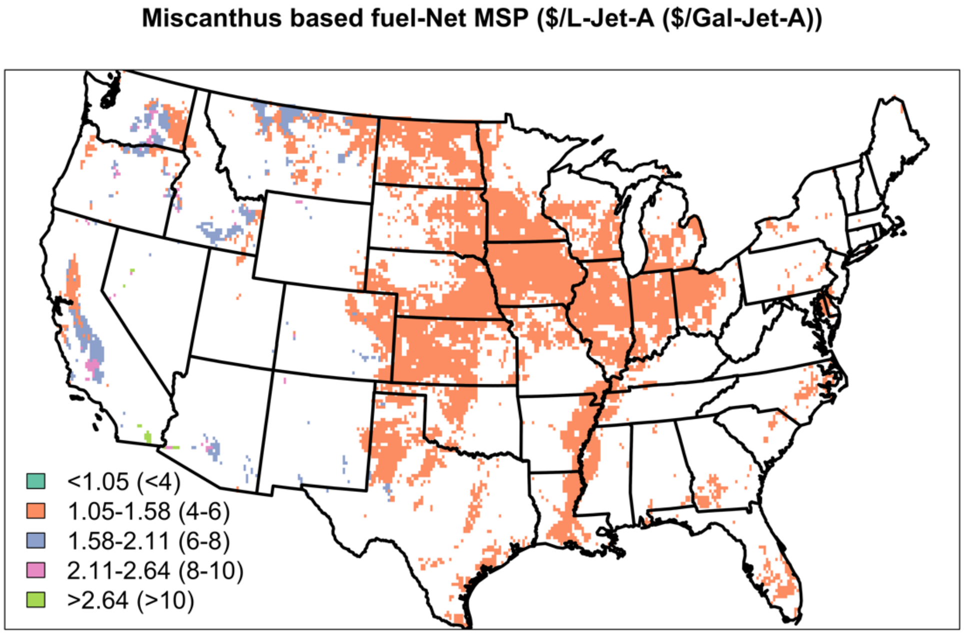

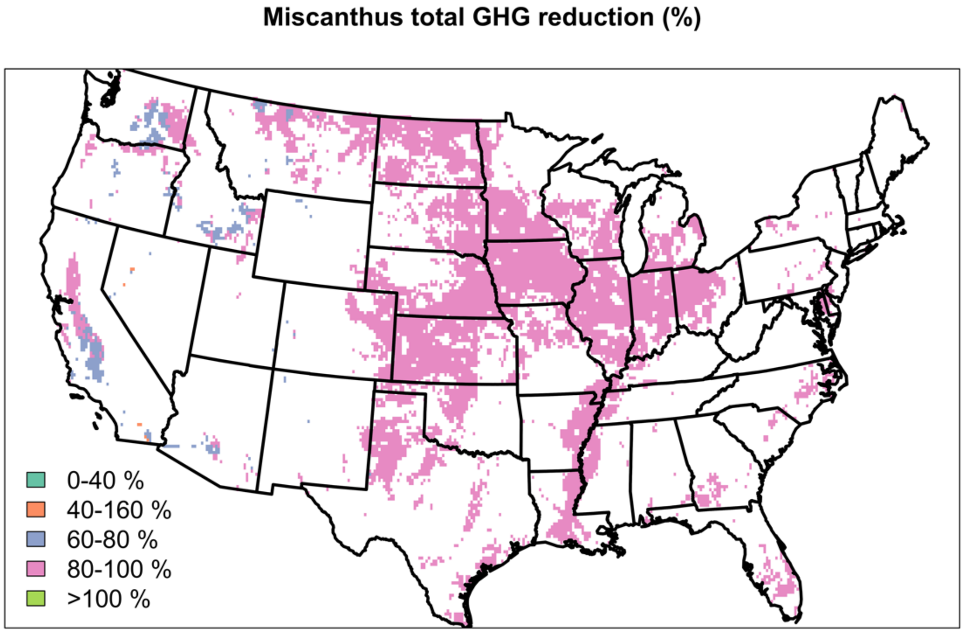

Fig. S11. Minimum selling price and carbon footprint of DMCO utilizing Miscanthus. The percent reductions in the carbon footprint of DMCO were calculated relative to the carbon footprint of conventional jet fuel of 89 gCO_{2e}/MJ(1)

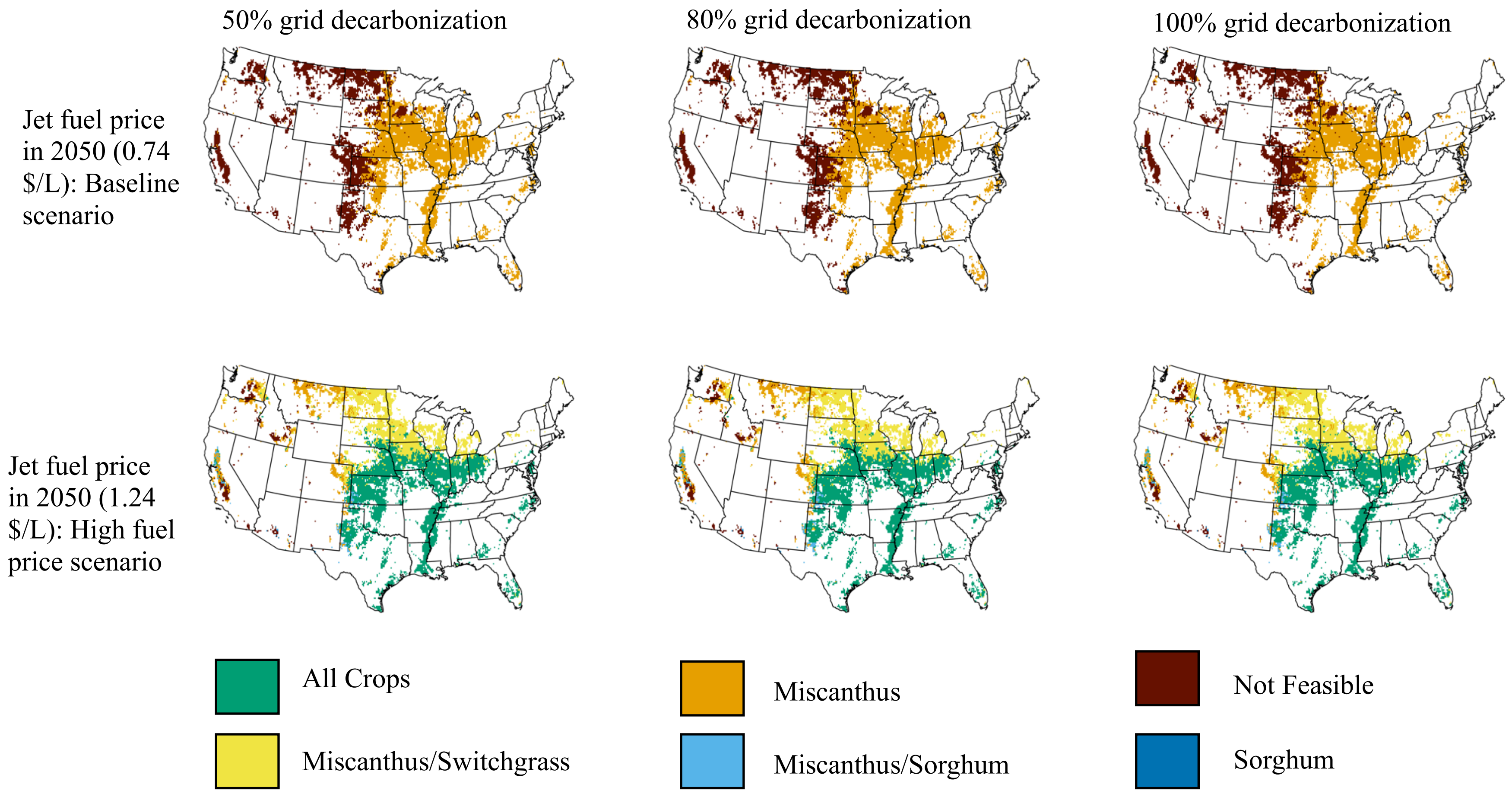

Fig. S12. Cost and carbon efficient bioenergy crops for the continental United States croplands with different carbon footprint of grid electricity; (left column) 50% grid decarbonization (center column) 80% grid decarbonization (right column) 100% grid decarbonization. Two rows show crop recommendation for two jet fuel price scenarios. This analysis considered carbon footprint of eGRID subregion electricity mix (Table S4).

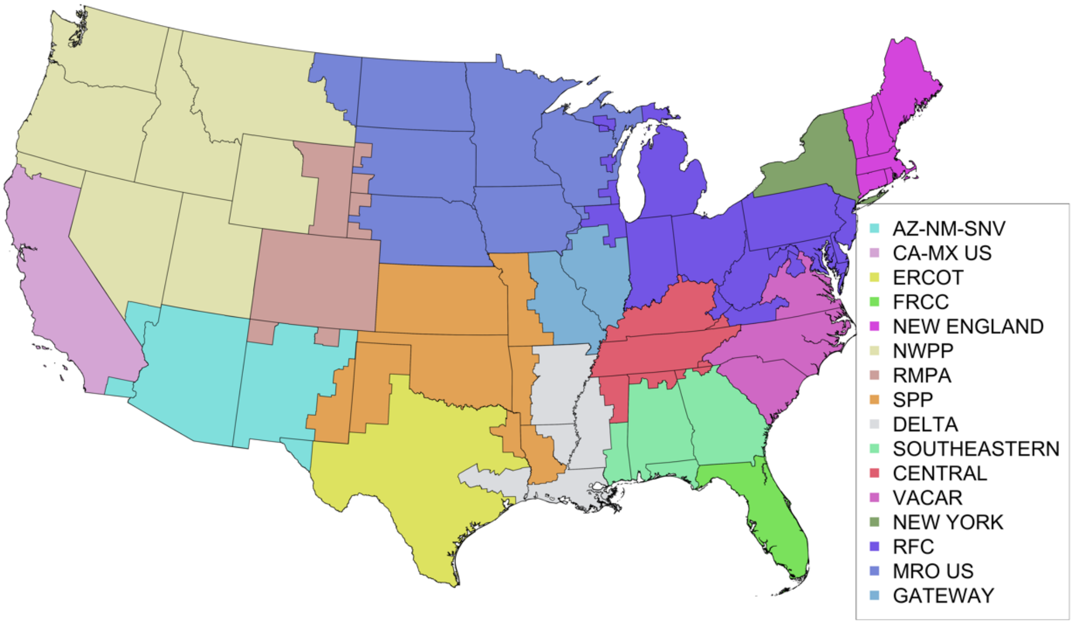

Figure S13. US EPA eGRID subregions used to calculate subregional electricity mixes.

Table S1. List of crop parameters for Miscanthus and Switchgrass.

Parameter name	Parameter value (Miscanthus)	Parameter value (Switchgrass)	Definition	Unit
PRDX(1)	3	2.75	Coefficient for calculating total potential production	g biomass m^{-2} $langley^{-1}$
PPDF(2)	50	45	Maximum temperature for production	°C
PPDF(3)	0.5	1	Right curve shape for parameterization of a Poisson Density Function curve to simulate temperature effect on growth	-
CFRTCN(1)	0.3	0.5	maximum fraction of C allocated to roots under maximum nutrient stress	-
CFRTCN(2)	0.2	0.3	minimum fraction of C allocated to roots with no nutrient stress	-
CFRTCW(1)	0.3	0.6	maximum fraction of C allocated to roots under maximum water stress	-
CFRTCW(2)	0.2	0.3	minimum fraction of C allocated to roots with no water stress	-
BIOMAX	113	200	biomass level above which the minimum and maximum C/E ratios of the new shoot increments equal pramn(1,2) and pramx(1,2) respectively	g biomass m^{-2}
PRAMN(1,1)	15	30	minimum C/N ratio with zero biomass	-
PRAMN(1,2)	30	90	minimum C/N ratio with biomass greater than or equal to BIOMAX	-
PRAMX(1,1)	35	50	maximum C/N ratio with zero biomass	-
PRAMX(1,2)	260	120	maximum C/N ratio with biomass greater than or equal to BIOMAX	-
PRBMN(1,1)	40	50	minimum C/N ratio for belowground matter	-
PRBMX(1,1)	60	55	maximum C/N ratio for belowground matter	-
FLIGNI(1,1)	0.05	0.02	lignin content of aboveground material	-
VLOSSP	0.15	0.1	fraction of aboveground plant N which is volatilized (occurs at harvest for crops or at senescence for grasses)	-
RDRJ	0.8	0.4	maximum juvenile fine root death rate at very dry soil conditions	day^{-1}
RTDTMP	-4	2	physiological shutdown temperature for root death and change in shoot/root ratio	°C
CRPRTF(1)	0.9	0.5	fraction of N transferred to a vegetation storage pool from grass/crop leaves at death	-
SNFXMX(1)	0.004	0	symbiotic N fixation maximum for grassland/crop	g N fixed g^{-1} C new growth
CURGDYS	75	120	number of days of unrestricted growth in a grass/crop system	days
CLSGRES	0.1	0.5	grass/crop late season growth restriction factor	-

S2. Biorefinery process modeling inputs

Table S2. Major input parameters used in process model that determines minimum selling price and greenhouse gas emissions

Parameter	Unit	Sorghum	Miscanthus	Switchgrass
**Lignocellulosic sugar feedstock**				
Mean biomass yield[β]	bdt/ha	9.7	14.6	7.6
Mean SOC sequestration[β]	$kgCO_{2e}/ha$	0.79	2.4	0.9
Mean N_2O emission[β]	$kgCO_{2e}/ha$	0.38	0.30	0.37
**Biomass composition(2, 3)**				
Cellulose	wt%	40	50.9	37.3
Hemicellulose	wt%	29.79	30.8	29.4
Lignin	wt%	9.89	10.8	18.7
**Biomass deconstruction(4, 5)**				
Solid loading rate	wt%	30	30	30
Ionic liquid loading rate	g/g-biomass	0.125	0.125	0.125
Ionic liquid cost	$/kg	1	1	1
Enzyme loading rate	mg/g-glucan	10	10	10
Enzyme cost	$/kg-protein	4	4	4
Cellulose to glucose	wt%	95	95	95
Xylan to xylose	wt%	90	90	90
**Bioconversion (6, 7)**				
Solid loading rate	wt%	25	25	25
Bioreactor power consumption	kW/m^3	0.11	0.11	0.11
Bioconversion time	h	36	36	36
Glucose utilization	%	95	95	95
Xylose utilization	%	85	85	85
Corn steep liquor loading	wt%	0.2	0.2	0.2
Diammonium phosphate loading	g/L	0.3	0.3	0.3
Corn steep liquor cost	$/kg	0.05	0.05	0.05
Diammonium phosphate cost	$/kg	0.3	0.3	0.3
**Recovery and separation (6, 7)**				
Isoprenol recovery	wt%	98	98	98
Ionic liquid recovery	wt%	99	99	99

Table S2. Contd.

Parameter	Unit	Sorghum	Miscanthus	Switchgrass
**Catalytic upgrading (6)**				
Steam loading rate	%	10.00	10.00	10.00
Phosphoric acid loading rate	%	5.00	5.00	5.00
Phosphoric acid cost	$/kg	0.60	0.60	0.60
Dimerization catalyst loading rate	wt%	0.0013	0.0013	0.0013
Dimerization catalyst loading cost	$/kg	7.14	7.14	7.14
Isoprenol to isoprene conversion rate	%	90.0	90.0	90.0
Isoprene to DMCOD isolated yield	%	89.00	89.00	89.00
Methylmagnesium chloride solution loading rate	wt%	0.26	0.26	0.26
Methylmagnesium chloride solution cost	$/kg	2.00	2.00	2.00
Hydrogen required[δ]	mol%	2.93	2.93	2.93
Hydrogen cost	$/kg	1.25	1.25	1.25
Raney Ni catalyst loading	wt%	0.43	0.43	0.43
Raney Ni catalyst cost	$/kg	10.49	10.49	10.49
DMCOD to DMCO isolated yield	wt%	98	98	98
**Wastewater treatment (8)**				
Organic matter to biogas	wt%	91	91	91
**Onsite energy generation (8)**				
Boiler chemicals cost	$/kg	4	4	4
Natural gas cost	$/kg	0.1	0.1	0.1

[β]Determined in a separate sugar model (Fig. S3).
[δ]Determined based on the molar ratio.

Table S3. Carbon footprint of major process chemicals and electricity considered in this study (6)

Component	Unit	Carbon footprint
Cholinium lysinate	$kgCO_{2e}/kg$	4.75
Corn liquor	$kgCO_{2e}/kg$	0.83
Diammonium phosphate	$kgCO_{2e}/kg$	1.15
Hydrogen	$kgCO_{2e}/kg$	6.30
Enzyme	$kgCO_{2e}/kg$	7.57
Natural gas	$kgCO_{2e}/kg$	2.69
Phosphoric acid	$kgCO_{2e}/kg$	0.56
Sodium hydroxide	$kgCO_{2e}/kg$	1.89
Sulfuric acid	$kgCO_{2e}/kg$	0.05
Tetrahydrofuran	$kgCO_{2e}/kg$	5.73
Nutrient for wastewater treatment	$kgCO_{2e}/kg$	0.83
Electricity	$kgCO_{2e}/kWh$	See Table S4
Iron-based dimerization catalyst	$kgCO_{2e}/kg$	0.89
Raney Ni catalyst	$kgCO_{2e}/kg$	6.87

Table S4. Greenhouse gas emission factors by eGRID subregion

Subregion acronym	Subregion[µ]	Output emission rates (lb CO_{2e}/MWh)(9)				Total GHG emissions ($kgCO_{2e}$/kWh)
		CO2	CH4	N2O	CO2e	
AKGD	ASCC Alaska Grid	1114.4	0.098	0.013	1120.8	0.508
AKMS	ASCC Miscellaneous	549.3	0.026	0.004	551.3	0.250
AZ-NM-SNV	WECC Southwest	952.3	0.068	0.01	956.9	0.434
CA-MX-USA	WECC California	453.2	0.033	0.004	455.3	0.207
ERCOT	ERCOT All	868.6	0.057	0.008	872.4	0.396
FRCC	FRCC All	861	0.055	0.007	864.5	0.392
HIMS	HICC Miscellaneous	1185.6	0.143	0.022	1195.6	0.542
HIOA	HICC Oahu	1694.5	0.185	0.028	1707.6	0.775
MROE	MRO East	1502.6	0.147	0.022	1512.6	0.686
MROW	MRO West	1098.4	0.119	0.017	1106.4	0.502
NEW ENGLAND	NPCC New England	488.9	0.077	0.01	493.8	0.224
NWPP	WECC Northwest	715.2	0.068	0.01	719.9	0.327
NYCW	NPCC NYC/Westchester	553.8	0.021	0.002	555.1	0.252
NYLI	NPCC Long Island	1209	0.157	0.02	1218.9	0.553
NYUP	NPCC Upstate NY	232.3	0.017	0.002	233	0.106
PRMS	Puerto Rico Miscellaneous	1537.3	0.084	0.013	1543.3	0.700
RFCE	RFC East	695	0.053	0.007	698.5	0.317
RFCM	RFC Michigan	1189.3	0.114	0.016	1197	0.543
RFCW	RFC West	1067.7	0.099	0.014	1074.4	0.487
RMPA	WECC Rockies	1242.6	0.117	0.017	1250.6	0.567
SPP-N	SPP North	1070	0.112	0.016	1077.6	0.489
SPP-S	SPP South	1002	0.07	0.01	1006.7	0.457
SRMV	SERC Mississippi Valley	806.8	0.043	0.006	809.6	0.367
SRMW	SERC Midwest	1584.4	0.169	0.025	1595.9	0.724
SRSO	SERC South	969.2	0.071	0.01	974	0.442
SRTV	SERC Tennessee Valley	949.7	0.087	0.013	955.6	0.433
SRVC	SERC Virginia/Carolina	675.4	0.058	0.008	679.1	0.308
U.S.	U.S. average	884.2	0.075	0.011	889.2	0.403

[µ]Figure S13 shows subregions.

S5. Density and calorific value

Table S1. Mass density and calorific value of selected methyl ketones and convention fuels

**Description**	**Density (kg/L)**	**Calorific value (MJ/kg)**	
		**Lower**	**Higher**
1,4-dimethylcyclooctane (DMCO)[1]	0.827	43.822	46.3[λ]
Jet-A/F-24[1]	>0.775	>42.8	46.2

[λ] Theoretically estimated value.

References

1. M. Wang (2007) Overview of GREET model development at Argonne. in *Center for Transportation Research, Argonne National Laboratory, GREET User Workshop, Argonne, IL*.
2. N. R. Baral *et al.*, Techno-economic analysis and life-cycle greenhouse gas mitigation cost of five routes to bio-jet fuel blendstocks. *Energy & Environmental Science* **12**, 807-824 (2019).
3. M. S. Roni, D. Thompson, D. Hartley, E. Searcy, Q. Nguyen, Optimal blending management of biomass resources used for biochemical conversion. *Biofuels, Bioproducts and Biorefining* **12**, 624-648 (2018).
4. H. D. Magurudeniya *et al.*, Use of ensiled biomass sorghum increases ionic liquid pretreatment efficiency and reduces biofuel production cost and carbon footprint. *Green Chemistry* **23**, 3127-3140 (2021).
5. E. Liu *et al.*, Understanding lignin fractionation and characterization from engineered switchgrass treated by an aqueous ionic liquid. *ACS Sustainable Chemistry & Engineering* **6**, 6612-6623 (2018).
6. N. R. Baral *et al.*, Production Cost and Carbon Footprint of Biomass-Derived Dimethylcyclooctane as a High-Performance Jet Fuel Blendstock. *ACS Sustainable Chemistry & Engineering* **9**, 11872-11882 (2021).
7. N. R. Baral *et al.*, Greenhouse gas footprint, water-intensity, and production cost of bio-based isopentenol as a renewable transportation fuel. *ACS Sustainable Chemistry & Engineering* **7**, 15434-15444 (2019).
8. D. Humbird *et al.*, Process design and economics for biochemical conversion of lignocellulosic biomass to ethanol. *Renew Energy* **303**, 147 (2011).
9. EPA (2019) eGRID Summary Tables 2019.
